# Ultralow Lattice Thermal Conductivity and High *ZT* of n-Type Polycrystalline SnSe Realized by Liquid Phase Sintering

**DOI:** 10.34133/research.0962

**Published:** 2025-10-21

**Authors:** Bin Su, Yilin Jiang, Hua-Lu Zhuang, Zhanran Han, Jincheng Yu, Haihua Hu, Jing-Wei Li, Hezhang Li, Yu-Xiao He, Lu Chen, Zhengqin Wang, Jing-Feng Li

**Affiliations:** ^1^ State Key Laboratory of New Ceramic Materials, School of Materials Science and Engineering, Tsinghua University, Beijing 100084, P. R. China.; ^2^ Fujian Science & Technology Innovation Laboratory for Optoelectronic Information of China, Fuzhou, Fujian 350108, P. R. China.; ^3^ State Key Laboratory of Functional Crystals and Devices, Fujian Institute of Research on the Structure of Matter, Chinese Academy of Sciences, Fuzhou 350002, P. R. China.; ^4^ Department of Precision Instrument, Tsinghua University, Beijing 100084, P. R. China.; ^5^ Department of Applied Physics, Graduate School of Engineering, Tohoku University, Sendai 980-8579, Japan.

## Abstract

SnSe has drawn increasing attention in thermoelectric applications because of its exceptional n/p-type characteristics. Although recent studies have reported an excellent figure of merit (*ZT*) value in p-type polycrystalline SnSe, achieving a breakthrough in thermoelectric performance for its n-type counterpart SnSe remains a critical challenge. The presence of V_Sn_ imposes a critical constraint on the synergistic optimization of carrier transport and phonon scattering in n-type SnSe. In this study, liquid phase sintering introduces high-density dislocations into n-type SnSe polycrystals, effectively scattering mid-frequency phonons. Huge lattice strain fluctuations caused by the defects enable an ultralow lattice thermal conductivity (0.21 W m^−1^ K^−1^) at 793 K. In addition, part of the liquid phase Sn tends to penetrate into the matrix, which leads to a higher carrier concentration and considerable enhancement in electrical properties. Consequently, a superior *ZT* (~1.9, 793 K) and an outstanding average *ZT* (*ZT*_ave_) (~0.72, 300 to 873 K) are achieved in polycrystalline SnSe, which rank at the top level reported for SnSe-based n-type thermoelectric materials, exceeding those of most n-type thermoelectric systems for mid-temperature applications.

## Introduction

On the basis of the Seebeck and Peltier effects, thermoelectric (TE) technology enables direct thermal–electrical energy conversion, demonstrating important potential for power generation and solid-state refrigeration [1,2]. The energy conversion efficiency of TE materials is mainly determined by the dimensionless figure of merit (*ZT*), which is expressed as *ZT* = *S*^2^*σT*/*κ*_T_, where *S*, *σ*, and *T* correspond to the Seebeck coefficient (*S*), electrical conductivity (*σ*), and absolute temperature (*T*), respectively, while thermal conductivity (*κ*_T_) combines the electronic thermal conductivity (*κ*_e_) and the lattice thermal conductivity (*κ*_L_) [3,4]. High-performance lead telluride and bismuth telluride are commercially employed for the fabrication of TE devices for applications in both the mid-temperature range and room-temperature range, respectively [5]. However, their usages are restricted by the toxicity and high cost of raw materials. Therefore, alternative TE materials made of economical, eco-friendly, and readily available elements, including chalcogenides, copper-based compounds, and Heusler compounds, have been explored recently [6–8].

Currently, SnSe has become the spotlight of attention due to its remarkable TE performance [9]. The *ZT* of SnSe single crystals for the p/n type can be optimized to over 2.5 [10,11]. However, for polycrystalline SnSe-based materials with lower production costs and superior mechanical properties, the TE properties are not as impressive as those of single crystals because of grain-boundary carrier scattering and the inevitable presence of SnO with a high thermal conductivity nature during the fabrication process [12]. Recently, the *ZT* of p-type SnSe polycrystals has been significantly enhanced via synergistic carrier and phonon engineering (*ZT* ≥ 2.0) [13]. Nevertheless, n-type SnSe polycrystals still lag behind single-crystal counterparts in TE performance. Basically, n-type polycrystalline SnSe can achieve a power factor (*PF*) comparable to that of p-type polycrystalline SnSe via chemical donor dopants, such as Cl, Br, I, Pr, and Nb, while its relatively high thermal conductivity does not match the low *κ*_T_ characteristics of single crystals [14]. This means that n-type polycrystalline SnSe still faces big challenges in the reduction of *κ*_T_ similar to its p-type counterpart. By introducing Sn vacancies (V_Sn_), the *κ*_T_ of p-type polycrystalline SnSe is significantly suppressed, as V_Sn_ induces lattice distortion and various high-dimensional defects, in addition to enhancing the electrical properties [15,16]. For n-type SnSe, V_Sn_ degrades carrier transport, and thus, suppressing its formation is critical. Additionally, introducing Se vacancies is challenging due to their high formation energy. [17]. Therefore, it is essential to seek other routes to optimize the *ZT* of n-type SnSe polycrystals. A critical question arises: can high-density defects be engineered to suppress *κ*_L_ while maintaining electrical properties?

Defect engineering, a strategy introducing multiscale defects, can suppress *κ*_L_ in materials [18]. Recently, the introduction of dislocations has been a popular route because the one-dimensional defect is able to scatter mid-frequency phonons with limited impact on carrier transport. High-density dislocations can be introduced by various methods, such as liquid phase sintering, melt centrifugation, annealing, and high-temperature sintering. For example, in the bismuth telluride system, high-density dislocations can be successfully constructed in the samples by pressure extrusion, liquid phase circulation sintering, and melt centrifugation, accounting for enhanced TE performance [19,20]. However, liquid phase sintering is rarely employed for fabricating SnSe-based materials. In theory, a liquid phase may also form during the sintering of SnSe powders with an excess of low-melting-point Sn, similar to the mechanism observed in bismuth telluride systems. Additionally, part of the liquid phase could react with the matrix during sintering, leading to compositional variations. With additional Sn atoms occupying V_Sn_, carrier transport is optimized.

**Fig. 1.**
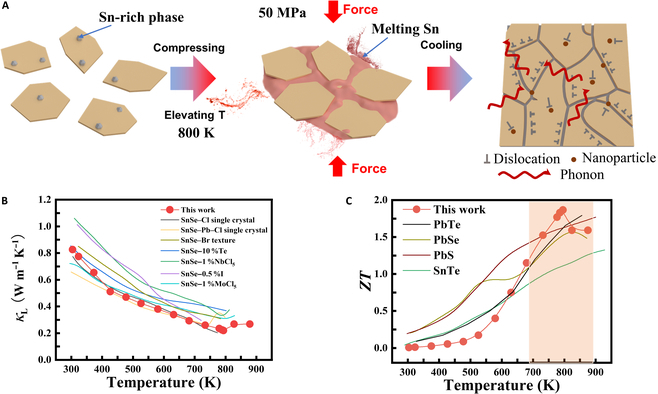
(A) Schematic diagram of liquid phase Sn extrusion sintering synthesis. (B) The comparison of lattice thermal conductivity with different materials working in n-type SnSe. (C) The comparison of figure of merit (*ZT*) with different materials working in the medium-temperature range.

In this work, an excess amount of Sn was added into SnSe (SnSe–Pb–Cl–*x*% Sn). At high temperatures, the excess Sn melted, acting as the liquid phase, part of which would be extruded from the matrix under a high pressure (Fig. 1A and Figs. S1 and S2). After liquid phase sintering, a large number of defects, such as dislocations and nanoprecipitates, were formed in the matrix. A remarkably low *κ*_L_ (~0.21 W m^−1^ K^−1^, 793 K) was obtained in the material, corresponding to the lowest reported value for n-type polycrystalline SnSe (Fig. 1B). Moreover, part of unextruded liquid phase Sn penetrated into the matrix during sintering, inhibiting the formation of intrinsic V_Sn_ that captures and scatters electrons and hence optimizing the electrical transport properties. At 793 K, *PF* reached a maximum of 6.3 μW cm^−1^ K^−2^. This n-SnSe polycrystal exhibits extraordinary *ZT* (~1.9, 793 K) and average *ZT* (*ZT*_ave_) (~0.72, 300 to 873 K). The high TE performance in the material reflects its potential to compete with other n-type TE materials for medium-temperature-range applications (Fig. 1C). This work establishes a novel pathway for promoting the TE performance in SnSe.

## Results and Discussion

### Electronic transport properties

**Fig. 2.**
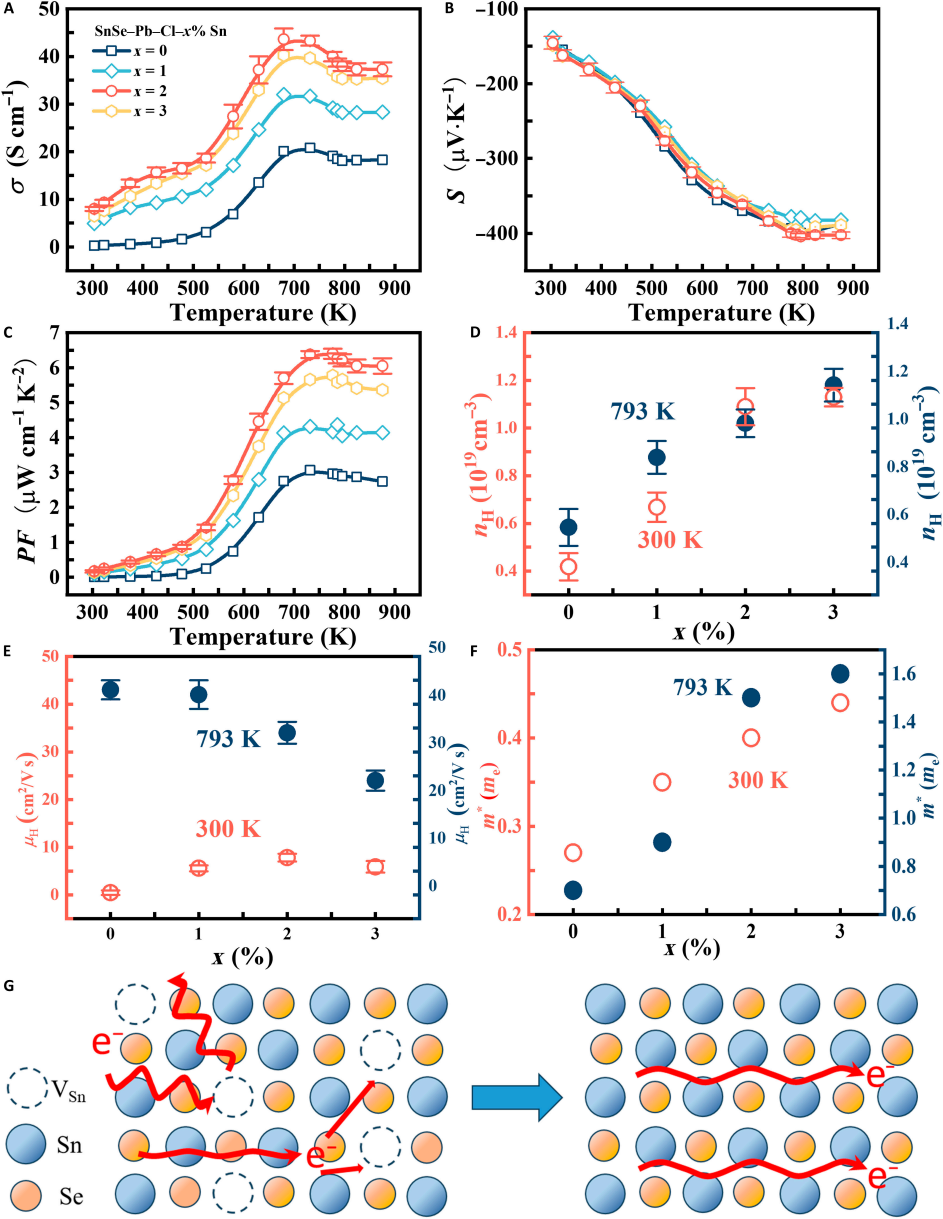
Temperature-dependent (A) *σ*, (B) *S*, and (C) power factor (*PF*) of SnSe–Pb–Cl–*x*% Sn. (D) The carrier concentration and carrier mobility of SnSe–Pb–Cl–*x*% Sn at 300 and 793 K. The m^*^ of SnSe–Pb–Cl–*x*% Sn at (E) 300 and (F) 793 K. (G) The schematic diagram of the vacancy compensation effect with SnSe–Pb–Cl–*x*% Sn samples.

The electrical transport performance can be significantly enhanced through liquid phase sintering. Figure 2A depicts the temperature dependence of the electrical conductivity of the samples measured parallel to the spark plasma sintering (SPS) pressure direction. The *σ* for all samples increases with increasing temperature before 650 K, showing a typical nondegenerate semiconducting behavior. Thermal excitation promotes carrier barrier crossing, while acoustic phonon scattering prevails at high temperatures. With excess Sn addition, *σ* gradually increases and peaks at ~43 S cm^−1^ in SnSe–Pb–Cl–2% Sn, significantly exceeding the ~20 S cm^−1^ of SnSe–Pb–Cl. Figure 2B shows characteristic *S*–*T* tendency in the materials, consistent with the literature [21]. Meanwhile, with increasing Sn content, Ι*S*Ι shows a downward trend followed by a rise; a maximum Ι*S*Ι value of ≈404 μV K^−1^ is achieved in SnSe–Pb–Cl–2% Sn at 793 K. Synergistic enhancement of *σ* and |*S*| achieves a superior *PF* (6.3 μW cm^−1^ K^−2^) in SnSe–Pb–Cl–2% Sn at 793 K (Fig. 2C).

To investigate the electrical properties of n-type SnSe polycrystals, the carrier transport of the samples was systematically analyzed (Fig. 2D and E). At 300 K, the Hall carrier concentration (*n*_H_) increases from 5.18 × 10^18^ cm^−3^ in SnSe–Pb–Cl to 1.02 × 10^19^ cm^−3^ in SnSe–Pb–Cl–2% Sn, with the corresponding Hall mobility (*μ*_H_) rising from 0.4 to 7.8 cm^2^ V^−1^ s^−1^. At 793 K, *n*_H_ still increases from 4.2 × 10^18^ to 1.09 × 10^19^ cm^−3^, while *μ*_H_ slightly decreases from 44 to 37 cm^2^ V^−1^ s^−1^. Then, at 300 and 793 K, the effective masses (m^*^) increase with increasing Sn content, from 0.27*m*_e_ to 0.44*m*_e_ and from 0.7*m*_e_ to 1.6*m*_e_, respectively (Fig. 2F and Fig. S3). Significant variations in the electronic structure of SnSe–Pb–Cl–*x*% Sn may be attributed to the changes in the matrix composition. According to electronic probe microscopic analysis, the signal of Sn in the matrix increases with increasing *x* (Table S2), reflecting elevated ratios of Sn to Se. As the liquid phase Sn shows better infiltration ability, it is more likely to be absorbed in the matrix during sintering. Part of excess Sn atoms may occupy the intrinsic V_Sn_, which can weaken defect scattering and the carrier capture effect (Fig. 2G) [22].

**Fig. 3.**
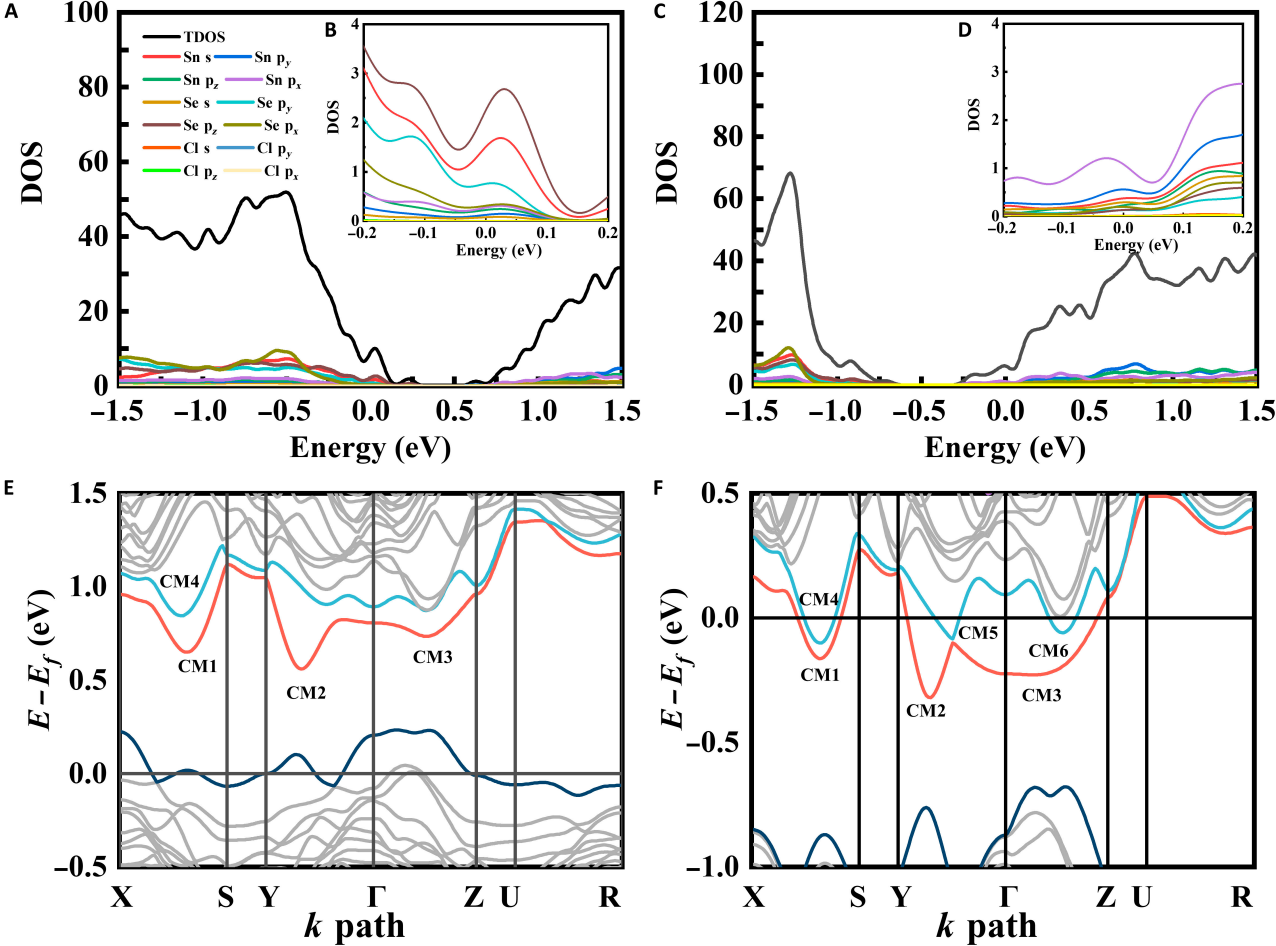
Electronic density of states (DOS) of (A and B) SnSe–Cl with V_Sn_ and (C and D) SnSe–Cl without V_Sn_. Band structures of (E) SnSe–Cl with V_Sn_ and (F) SnSe–Cl without V_Sn_.

Electronic structure calculations were conducted to investigate V_Sn_’s impact on charge transport, specifically the significantly increased *n*_H_ and |*S*| values. Given that low-concentration isovalent Pb doping does not significantly alter the Fermi level, the SnSe–Cl system was selected as a model to achieve both consistency with experimental results and accuracy in the simulated conclusions. Figure 3A to D illustrate the corresponding density of states for SnSe. The valence band maximum of SnSe is predominantly constituted by Se 4p*_z_* orbitals, while the conduction band minimum shows predominant contributions from Sn 5p*_x_* orbitals. This distinct orbital participation at band edges suggests that the Sn/Se ratio could significantly modulate the electronic properties of SnSe. Compared to that of SnSe–Cl with V_Sn_, the Fermi level of SnSe–Cl without V_Sn_ moves down into the 6 primary conduction bands (Fig. 3E and F). This indicates that the filling of V_Sn_ pushes the Fermi level into the conduction band, making SnSe an n-type semiconductor. Therefore, the Fermi level shifts deeply into the conduction bands due to the reduced V_Sn_, leading to a higher *n*_H_. Then, the energy offset between CM2 and CM3 (positioned between the Γ and Y points) diminishes, and the width of CM3 expands, resulting in heightened band degeneracy and effective mass and hence an elevated |*S*| value. Despite a lower *n*_H_, the SnSe–Pb–Cl sample exhibits a relatively low |S| at room temperature due to enhanced carrier scattering from nanoparticles in SnSe–Pb–Cl–*x*% Sn. As the temperature rises, phonon scattering dominates and band structure effects strengthen, leading to a higher |*S*| in SnSe–Pb–Cl between 480 and 680 K. At further elevated temperatures, increased band degeneracy and effective mass in SnSe–Pb–Cl–*x*% Sn samples cause the |*S*| of both systems to converge. An acceleration of the carrier movement leads to a higher frequency of collisions between carriers, resulting in an additional scattering effect at high temperature. As the carrier concentration increases at high temperatures, the negative effect on carrier mobility becomes evident, indicating that *μ*_H_ decreases with increasing Sn content. Consequently, in comparison to those of the unextruded sample, the *σ* and *PF* of the samples with excess Sn are markedly increased over the entire temperature range.

### Thermal transport properties

**Fig. 4.**
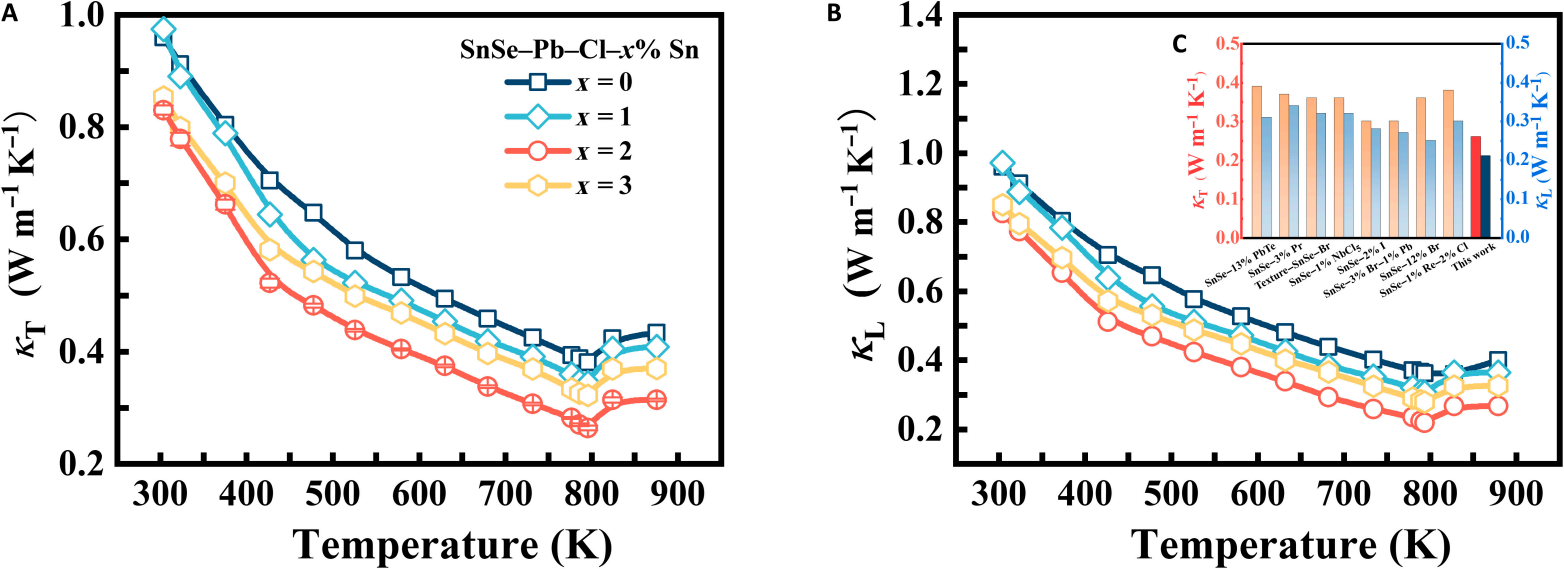
Temperature-dependent (A) *κ*_T_ and (B) *κ*_L_ for SnSe–Pb–Cl–*x*% Sn. (C) The minimum *ZT* value and the average *ZT* value of n-type SnSe polycrystals in this work with those in previous studies.

Figure 4A and B show the variations of *κ*_T_ and *κ*_L_ for samples aligned with the SPS pressure direction. All samples display decreasing *κ*_T_ and *κ*_L_ with temperature until reaching 823 K. However, *κ*_T_ exhibits an increase at 823 K, resulting from bipolar effects induced by the structural transition [23]. A low Pb content does not significantly affect the material’s phase transition temperature, which is consistent with the unchanged inflection point in thermal conductivity. In general, *κ*_T_ and *κ*_L_ decrease gradually with increasing Sn content. Surprisingly, the thermal conductivity of SnSe–Pb–Cl–2% Sn retains a low level compared to those of its counterparts; minimum *κ*_T_ and *κ*_L_ of 0.26 and 0.21 W m^−1^ K^−1^ are achieved at 793 K, respectively, which are ultralow values compared to the data for reported n-type polycrystalline SnSe (Fig. 4C) [17,24–30]. The ultralow *κ*_L_ nearly reaches the theoretical minimum value of SnSe. When the tin content exceeds 3%, the excess tin fills Sn vacancies, increases *n*_H_, reduces point defect scattering, and may lead to the formation of highly thermally conductive Sn nanoparticles, collectively resulting in an increase in *κ*_T_.

### Overall figure of merit

**Fig. 5.**
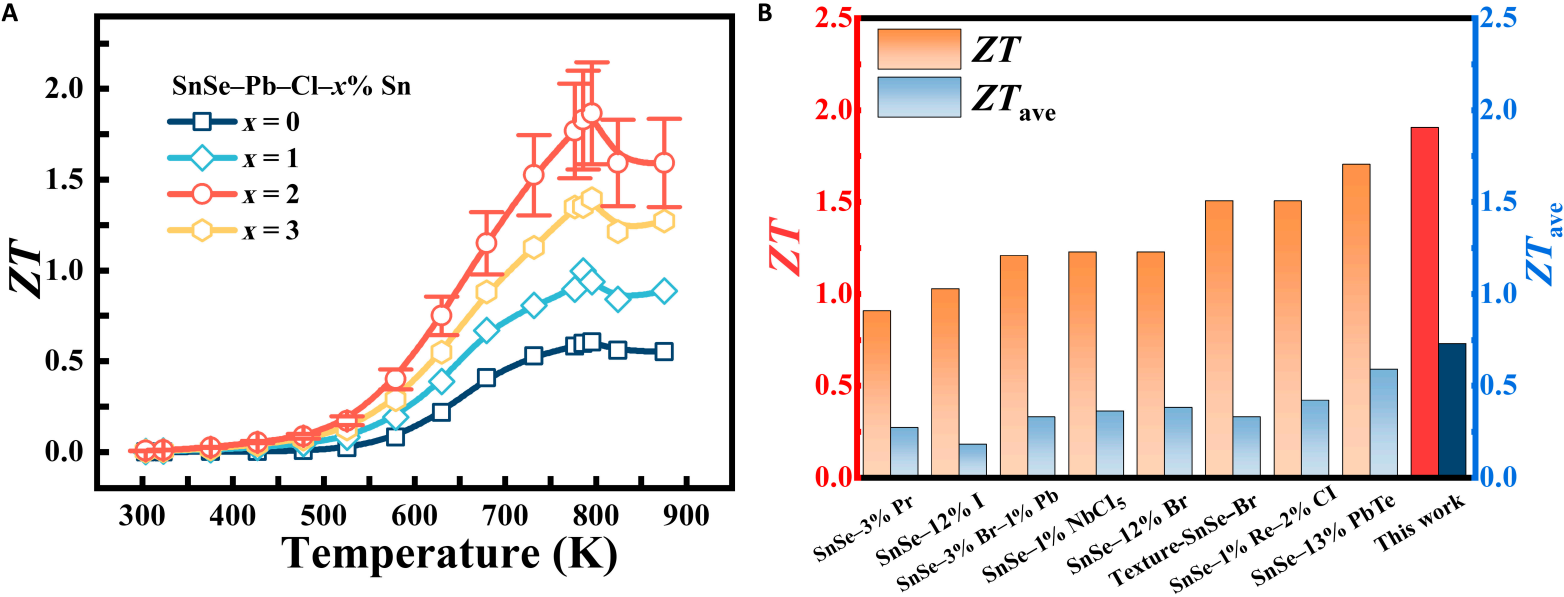
(A) Figure of merit *ZT* as a function of temperature for SnSe–Pb–Cl–*x*% Sn. (B) Comparison of maximum *ZT* and *ZT*_ave_ of SnSe–Pb–Cl–*x*% Sn with those of other reported n-type polycrystalline SnSe.

Figure 5A shows the temperature-dependent *ZT* of n-type SnSe samples. SnSe–Pb–Cl–2% Sn attains a remarkable *ZT* ~ 1.9 at 793 K, due to the synergistic optimization of electronic and phonon transport. The reproducible electrical/thermal transport data confirm outstanding data fidelity in this high-*ZT* material (Fig. S4). Furthermore, a remarkable *ZT*_ave_ of 0.72 between 300 and 873 K achieves a 12.2% theoretical *η*_max_. The highest *ZT* and *ZT*_ave_ in this work represent state-of-the-art values among n-type polycrystalline SnSe systems (Fig. 5B) [17,24–27,29–31].

### Microstructural analysis

**Fig. 6.**
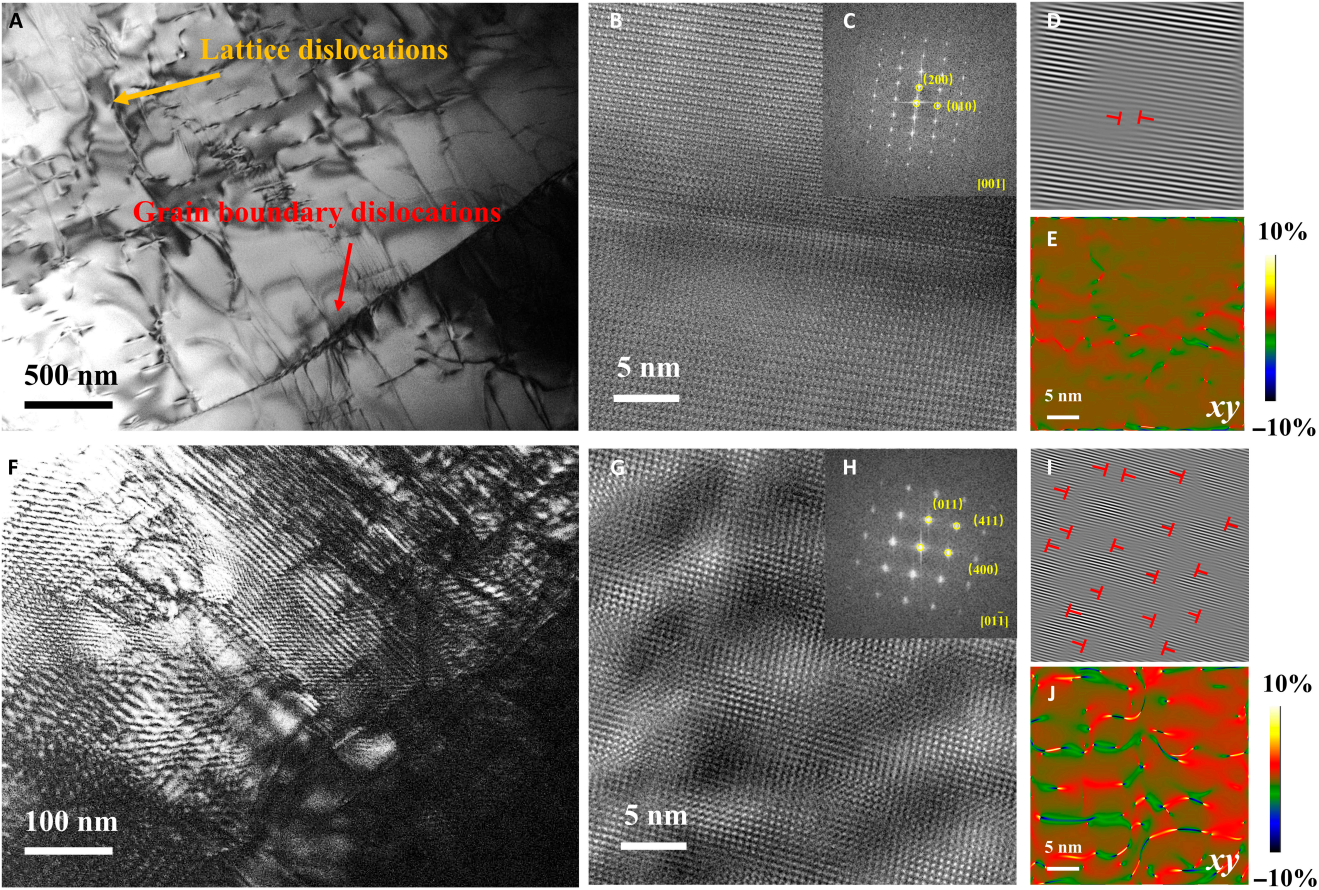
(A) Low-magnification transmission electron microscopy (TEM), (B) high-resolution transmission electron microscopy (HRTEM), (C) fast Fourier transform (FFT), (D) inverse fast Fourier transformation (IFFT), and (E) geometric phase analysis (GPA) images of the dislocation of SnSe–Pb–Cl–2% Sn. (F) Low-magnification TEM image, (G) HRTEM, (H) FFT, (I) IFFT, and (J) GPA images of the dislocation networks of SnSe–Pb–Cl–2% Sn.

To further study the outstanding TE performance of samples with excess Sn, the SnSe–Pb–Cl–2% Sn sample was investigated by transmission electron microscopy (TEM). The matrix and grain boundaries contain a high density of dislocations (~2.3 × 10^12^ cm^−2^), while few defects are observed in the pure polycrystalline SnSe–Pb–Cl (Fig. 6A to D and Fig. S5). In addition, Fig. 6F shows a typical dislocation network, and the corresponding high-resolution TEM image (Fig. 6G) depicts that the high-density dislocations (~6.8 × 10^13^ cm^−2^) are linked together to form these dislocation networks. The inverse fast Fourier transformation and fast Fourier transform images also show many extra half-planes of those dislocations that appear in fine grains. The strain distribution obtained via geometric phase analysis reveals pronounced lattice deformation surrounding dislocations within the matrix (Fig. 6E and J and Figs. S6 and S7). Surprisingly, a huge strain fluctuation (~10%) caused by dislocation networks is observed, as a consequence of the intersection of multiple dislocation lines. The high-density dislocations, acting as high-dimensional crystalline defects, can effectively enhance phonon scattering while remarkably preserving charge carrier mobility in the material [32]. Additionally, as shown in Figs. S8 and S9, there are several nanostructured regions observed in the matrix. High-density dislocations are around the nanostructured regions. Then, as shown in the high-resolution TEM image, many lattice distortions were found in the nanostructured region (~4.5 × 10^13^ cm^−3^) (Fig. S9B and D). According to the geometric phase analysis, significant average lattice strain fluctuations were observed in this nanostructured region, indicating that these defects can perform as phonon scattering centers (Fig. S9E). The energy-dispersive x-ray spectroscopy analysis revealed a higher proportion of the Sn element in the nanostructured region (Fig. S9C). We consider this region as the Sn-rich SnSe phase.

Liquid phase Sn originates from the monotectic phase microplate; the final reaction product is composed of Sn and SnSe nanoparticles, as indicated by the monotectic transition reaction formula $L_{c}\to S_{\text{SnSe}}+L_{\mathrm{Sn}}$ (Figs. S10 to S14). During liquid phase sintering, the wetting liquid phase infiltrates grain boundaries, where the enhanced atomic mobility in the liquid state results in significantly higher diffusivity compared to the case under solid-state conditions, facilitating compositional changes in SnSe. The liquid phase wets and encapsulates the solid particles, driving grain rearrangement via capillary forces. Meanwhile, additional pressure from the liquid phase compaction process facilitates dislocation formation and further accelerates grain rearrangement, resulting in dislocation networks at the grain boundaries [33,34]. It is hypothesized that the residual Sn is retained within the liquid phase extrusion zone, leading to the formation of nanostructured regions. Compositional differences between the Sn-rich nanoparticles and the matrix induce lattice mismatch, which promotes interfacial dislocation generation during cooling to release the accumulated strain energy [35]. Moreover, the high-density dislocations surrounding nanostructured regions also arise from the differential thermal expansion coefficient (TEC) of the Sn-rich nanoparticles and the host material. Based on Eshelby’s inclusion theory, the misfit strain *ε* can be determined by Eq. (1) [36]:

$$\varepsilon =\left(a_{m}-a_{l}\right)\times ∆T$$ (1)

where *a_m_* and *a_l_* denote the TECs of the matrix and inclusions, respectively, and A*T* represents the temperature change. Notably, SnSe exhibits a strongly anisotropic TEC, including the orientations with negative values (around 50.5 × 10^−6^ K^−1^ [in the *a* axis], 35.8 × 10^−6^ K^−1^ [in the *b* axis], and −21.1 × 10^−6^ K^−1^ [in the *c* axis] at 325 to 800 K). This may contrast markedly with the TEC behavior of Sn-rich nanoparticles (~23 × 10^−6^ K^−1^) [37,38].

The liquid phase temperature (~500 K) is much lower than the sintering temperature (~800 K), indicating that dislocations caused by the liquid phase are formed before grain growth occurs, and some of these dislocations will be integrated into the grain as a result of grain growth. Consequently, liquid phase sintering helps to generate a high density of defects that are capable of effectively scattering phonons, thereby leading to a drastic reduction in both *κ*_T_ and *κ*_L_. Moreover, since the phase structure, texturing degree (0.61, 0.63, 0.62, and 0.56), grain morphology, and grain size do not show significant changes during liquid phase sintering (Figs. S15 to S17), it is inferred that microstructure variation should be responsible for the decreased *κ*_L_.

Based on the Debye–Callaway model, we accurately simulated the *κ*_L_ in SnSe–Pb–Cl–2% Sn polycrystals, showing high-temperature consistency with experimental data (Fig. S18A). At low temperatures, the predicted value of *κ*_L_ may deviate from the experimentally obtained value calculated with acoustic phonon scattering assumption attributed to the complex scattering mechanism. Phonon scattering exhibits wavelength-dependent selectivity: long-wavelength phonons are dominated by grain boundaries, short-wavelength phonons are attributed to point defects, and mid-wavelength phonons are scattered by dislocations and precipitates. The interplay of these mechanisms drives a pronounced *κ*_L_ reduction (Fig. S18B).

## Conclusion

This work demonstrates a remarkably high TE performance in n-type polycrystalline SnSe through liquid phase sintering. Significantly, the incorporation of excess Sn inhibits the formation of intrinsic V_Sn_, simultaneously optimizing both *σ* and *S*. The liquid phase sintering process also introduces substantial dislocations, resulting in a large number of strain fluctuations that scatter phonons. Polycrystalline SnSe–Pb–Cl–2% Sn achieves superior *ZT* of 1.9 at 793 K and *ZT*_ave_ of 0.72 from 300 to 873 K, attributed to the optimization of electron–phonon transport. This work confirms liquid phase sintering as an effective strategy for decoupling electron–phonon transport in TE materials.

## Methods

### Synthesis

The starting materials of Sn, Se, Pb, and SnCl_2_ were weighed in stoichiometric ratios of Sn_1+*x*_Pb_0.02_Se_0.94_Cl_0.06_ (*x* = 0%, 1%, 2%, and 3%). The samples with different compositions (Sn_1+*x*_Pb_0.02_Se_0.94_Cl_0.06_ [*x* = 0%, 1%, 2%, and 3%]) were denoted as SnSe–Pb–Cl–*x*% Sn (*x* = 0, 1, 2, and 3). Doping concentrations of 2% for Pb and 6% for Cl represent the optimal composition for this study (Figs. S19 and S20). The starting materials were weighed and loaded into a 10-mm-diameter quartz tube under an inert atmosphere in a glove box. This tube was then sealed inside a 13-mm-diameter outer quartz tube under a high vacuum (<3 × 10^−3^ Torr). The double-sealed ampoule was vertically placed in a box furnace, heated to 1,223 K, and held at that temperature for 8 h, followed by furnace cooling to room temperature. The resulting ingots were cleaned and mechanically ground into fine powders These powders were then loaded into a graphite die and densified via SPS (SPS 211Lx, Fuji Electronic, Japan) at 823 K for 5 min under an axial pressure of 50 MPa. The heating rate to the target temperature was 25 min, after which the sample was allowed to cool naturally while the pressure was maintained throughout the entire SPS process.

### Characterization

Phase identification was conducted by x-ray diffraction (SmartLab, Rigaku, Japan) using Cu Kα radiation (*λ* = 1.5418 Å). The surface morphology and microstructural features were characterized using field-emission scanning electron microscopy (Zeiss Merlin, Germany) and TEM (JEOL 2100F, Japan). Elemental distribution mappings were acquired through electron probe microanalysis electronic probe microscopic analysis (JEOL JXA-8230, Japan). Grain size, inverse pole figures, and local crystallographic orientation were analyzed via electron backscatter diffraction (Oxford Instruments, UK).

### Transport property measurement

Both *σ* and *S* were measured by a ZEM-3 system (Ulvac-Riko, Japan). The thermal diffusivity (*D*) of the samples was determined using the laser flash method on an LFA 457 instrument (Netzsch, Germany). *κ*_T_ can be obtained via the equation *κ*_T_ = *DC*_p_*d*, where *C*_p_ derives from the Dulong–Petit limit and *d* is measured by the Archimedes method (Table S1, Supplementary Materials). *n*_H_ and *μ*_H_ were measured using the van der Pauw method (ResiTest8340DC, Tokyo Instruments, Japan).

### The DFT calculation

Density functional theory (DFT) calculations utilized the plane-wave pseudopotential approach in the Vienna Ab initio Simulation Package [39,40]. Projector augmented wave pseudopotentials model electron–ion interactions [41]. The Perdew–Burke–Ernzerhof generalized gradient approximation accounts for exchange-correlation effects [42]. Electronic structure and density of states calculations utilized a 5 × 7 × 5 Monkhorst–Pack mesh for Brillouin zone sampling [43]. Supercells were constructed based on the *Pnma* primitive cell using a 1 × 2 × 2 expansion. Initial models for electronic structure calculations consisted of a 64-atom supercell (32 Sn, 31 Se, and 1 Cl) for the system with V_Sn_ and a 63-atom supercell (31 Sn, 31 Se, and 1 Cl) for the system without V_Sn_. Both supercells were fully relaxed (unit cell parameters and atomic positions) before performing band structure calculations. Calculations used a 450-eV plane-wave cutoff energy. Ionic relaxation proceeded until atomic forces were <0.01 eV/Å.

## Data Availability

All data are available in the paper or the Supplementary Materials.

## Supplementary Materials

Supplementary 1 Figs. S1 to S20 Tables S1 and S2 Supplementary Notes
