# Supplementary material for: Ultralow Lattice Thermal Conductivity and High ZT of n-Type Polycrystalline SnSe Realized by Liquid Phase Sintering
Source: Research (Wash D C). 2025 Oct 21;8:0962. doi: 10.34133/research.0962 (PMC12538152; doi:10.34133/research.0962)
Supplement: Supplementary 1 — Figs. S1 to S20 Tables S1 and S2 Supplementary Notes [file research.0962.f1.docx]

**Supporting Information**

**Ultralow Lattice Thermal Conductivity and High *ZT* of n-Type Polycrystalline SnSe Realized by Liquid Phase** **Sintering**

Bin Su,^1,2,3^ Yilin Jiang,^1^ Hua-Lu Zhuang,^1^ Zhanran Han,^1^ Jincheng Yu,^1^ Haihua Hu,^1^ Jing-Wei Li,^1^ Hezhang Li,^1,4^ Yu-Xiao He,^1^ Lu Chen,^1^ Zhengqin Wang^1^ and Jing-Feng Li^1,5^*

^1^ State Key Laboratory of New Ceramics and Fine Processing, School of Materials Science and Engineering, Tsinghua University, Beijing 100084, P. R. China

^2^ Fujian Science & Technology Innovation Laboratory for Optoelectronic Information of China, Fuzhou, Fujian 350108, P. R. China

^3^ State Key Laboratory of Functional Crystals and Devices, Fujian Institute of Research on the Structure of Matter, Chinese Academy of Sciences, Fuzhou, 350002, P. R. China

^4^ Department of Precision Instrument, Tsinghua University, Beijing, 100084, P. R. China

^5^ Department of Applied Physics, Graduate School of Engineering, Tohoku University, Sendai, 980-8579, Japan

Corresponding author: Email: jingfeng@mail.tsinghua.edu.cn

**Keywords:** thermoelectric; tin selenide; liquid phase sintering; electrical transport; dislocations.


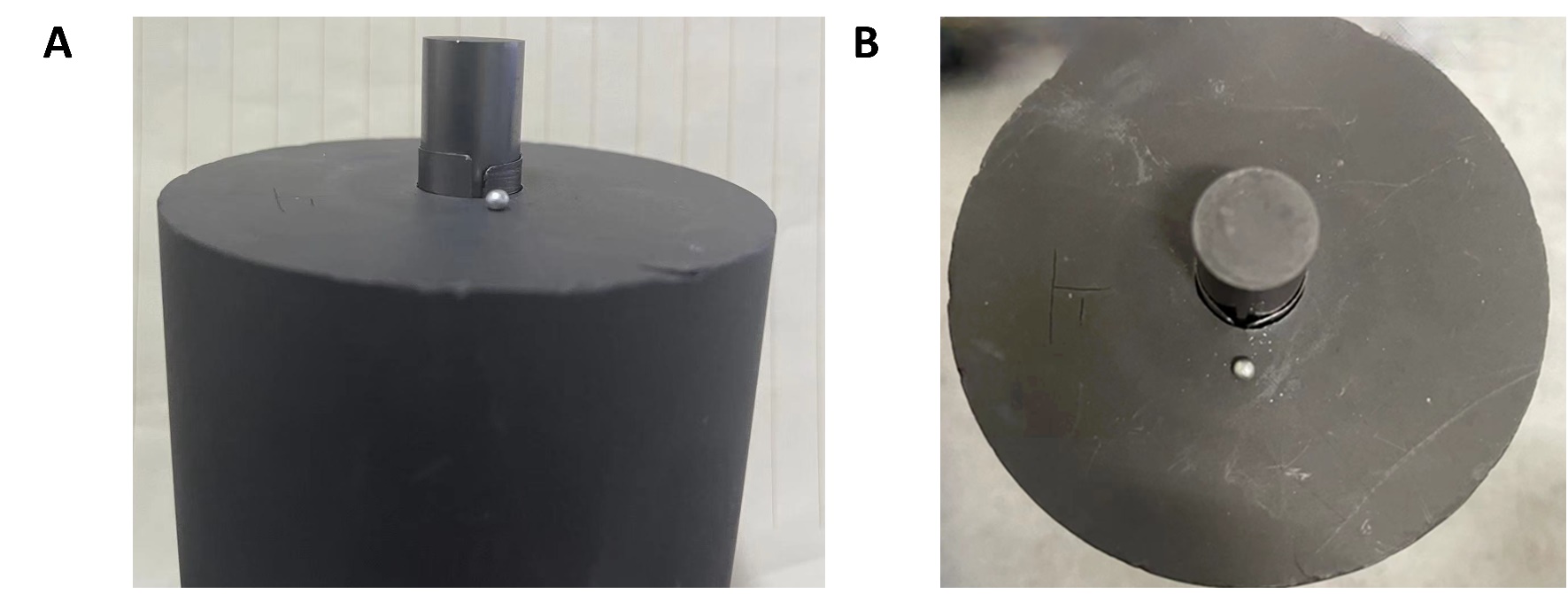


**Fig. S1** Pictures after Liquid phase extrusion sintering.


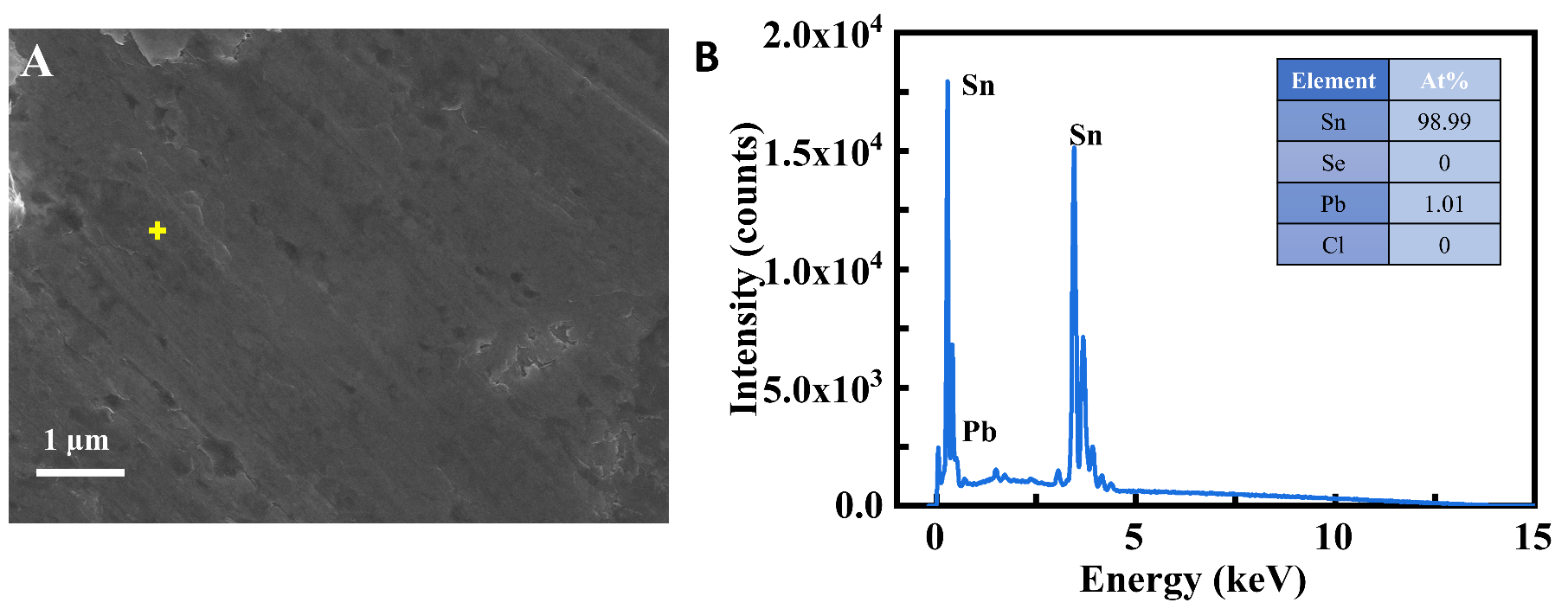


**Fig. S2** (A) SEM images and (B) EDS of the extruded particle.


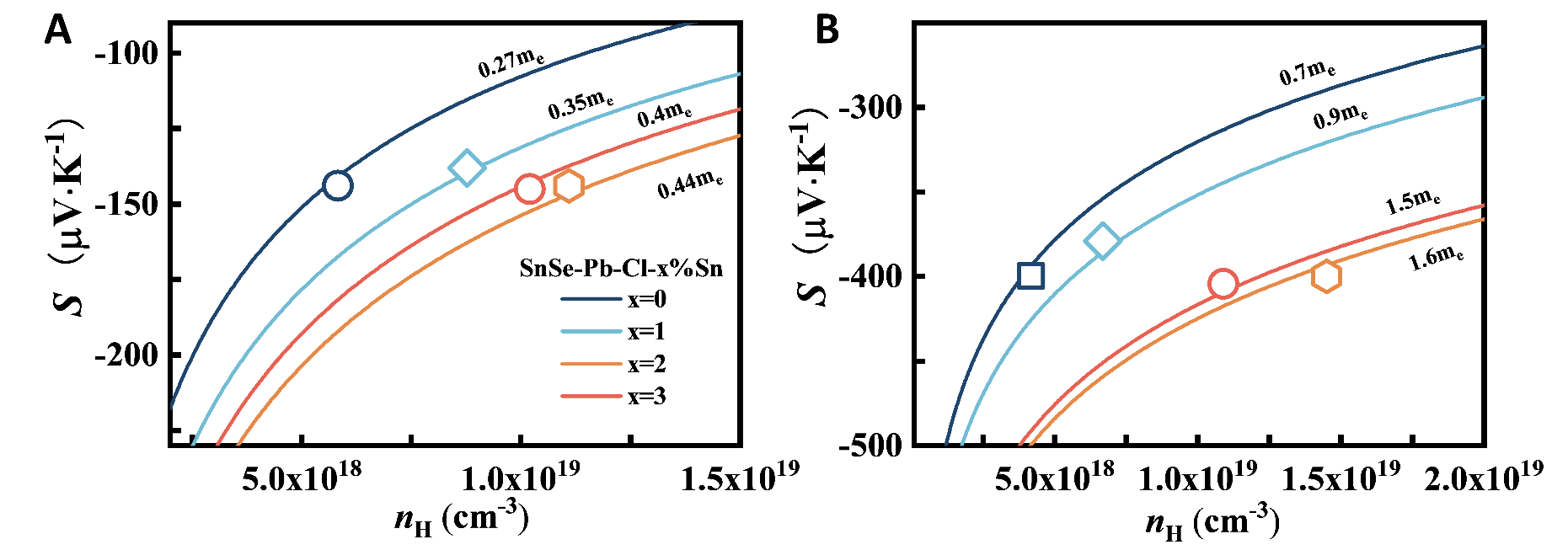


**Fig. S3** *S* as a function of *n*_H_ for SnSe-Pb-Cl-x%Sn at 300 K and 793 K.


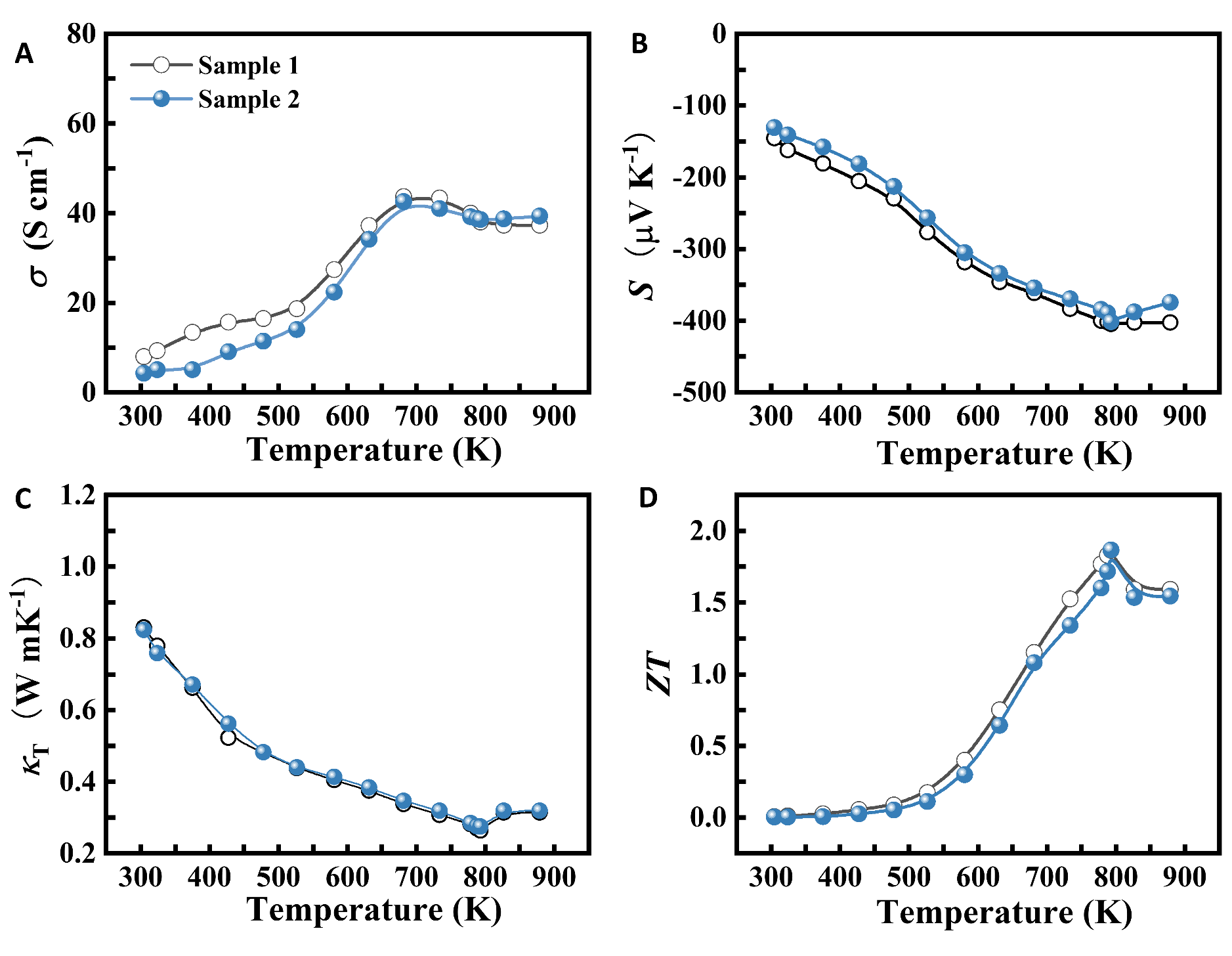


**Fig. S4** Repeat measurement results of (A) electrical conductivity, (B) Seebeck coefficient, (C) thermal conductivity, (D) *ZT* for SnSe-Pb-Cl -2%Sn.


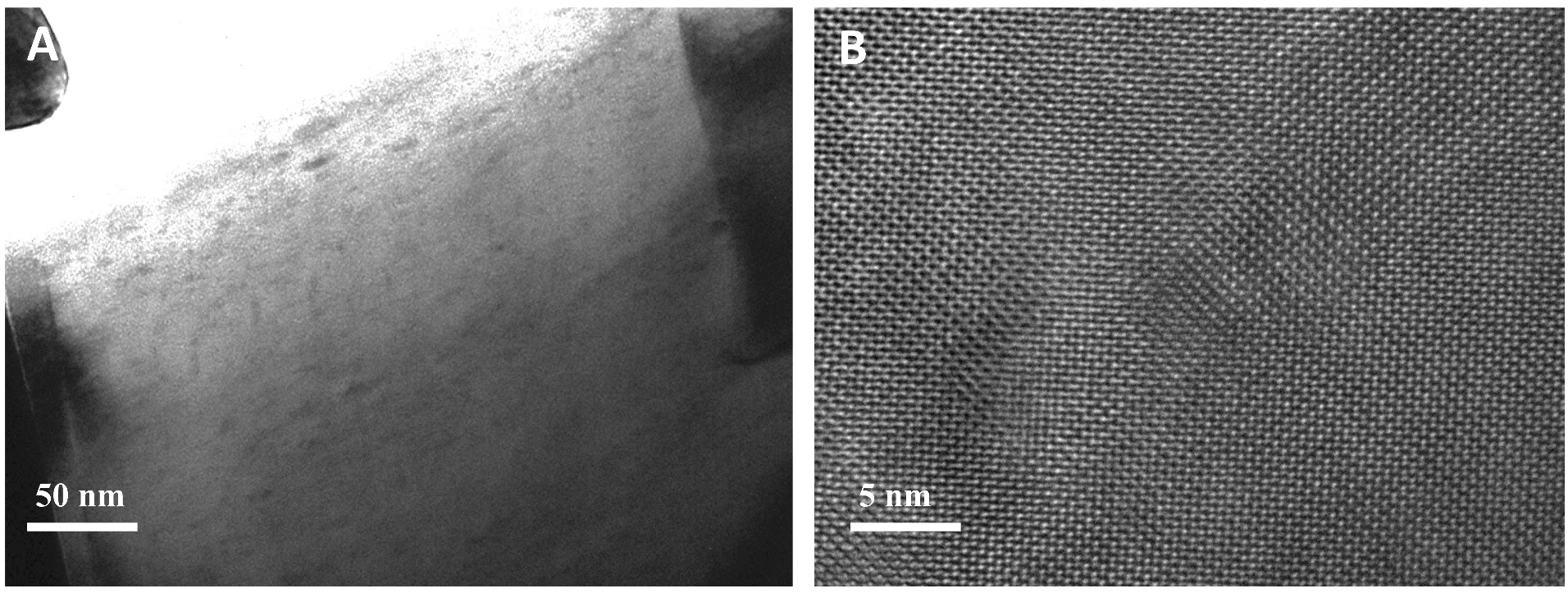


**Fig. S5** (A)TEM and (B) HRTEM images of SnSe-Pb-Cl-2%Sn.


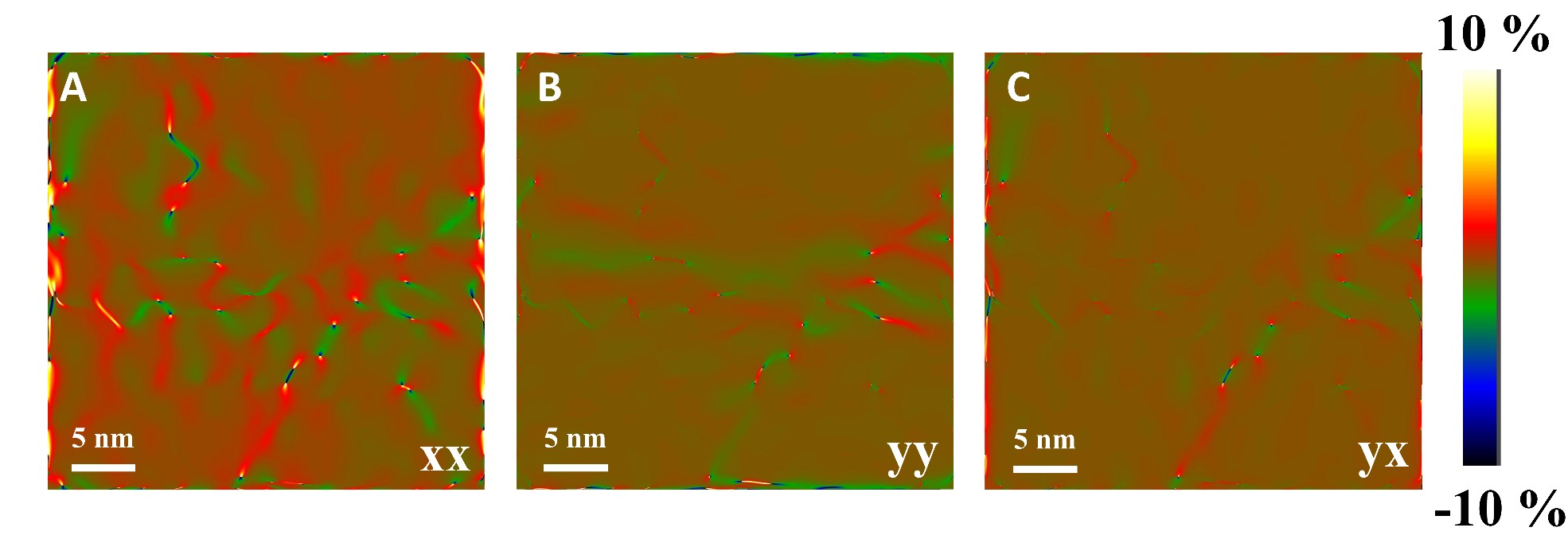


**Fig. S6** Lattice strain maps for dislocations of SnSe-Pb-Cl-2%Sn along different directions.


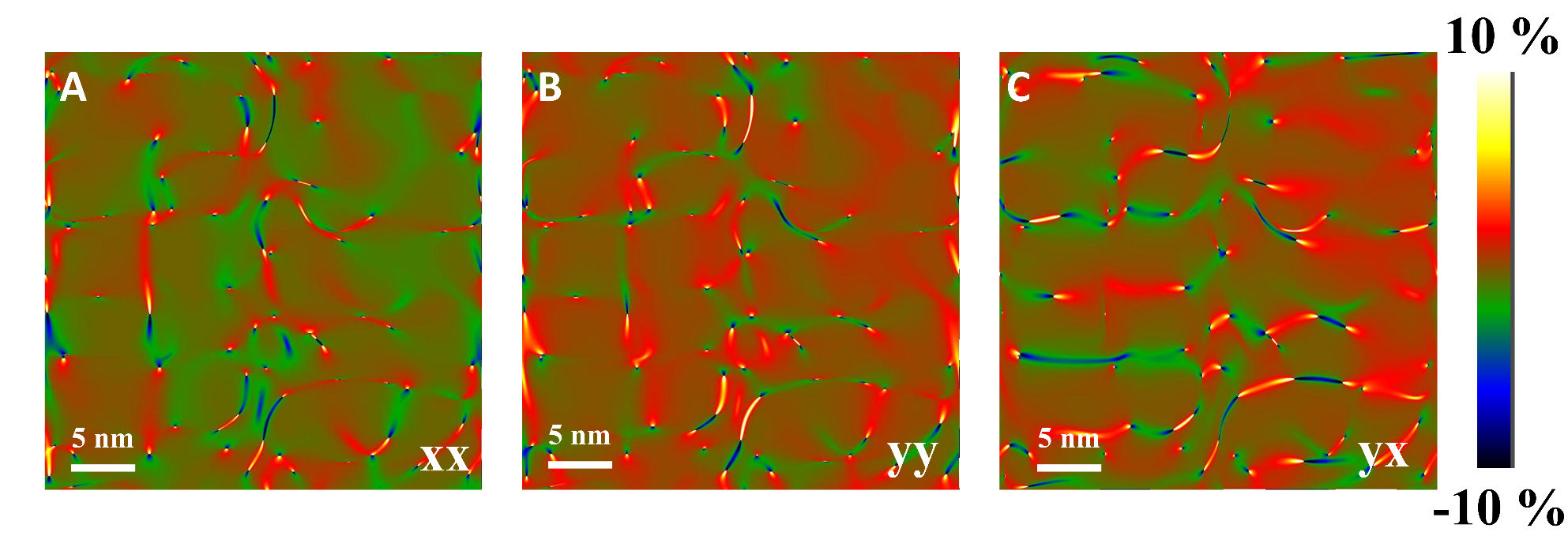


**Fig. S7** Lattice strain maps for dislocations networks of SnSe-Pb-Cl-2%Sn.


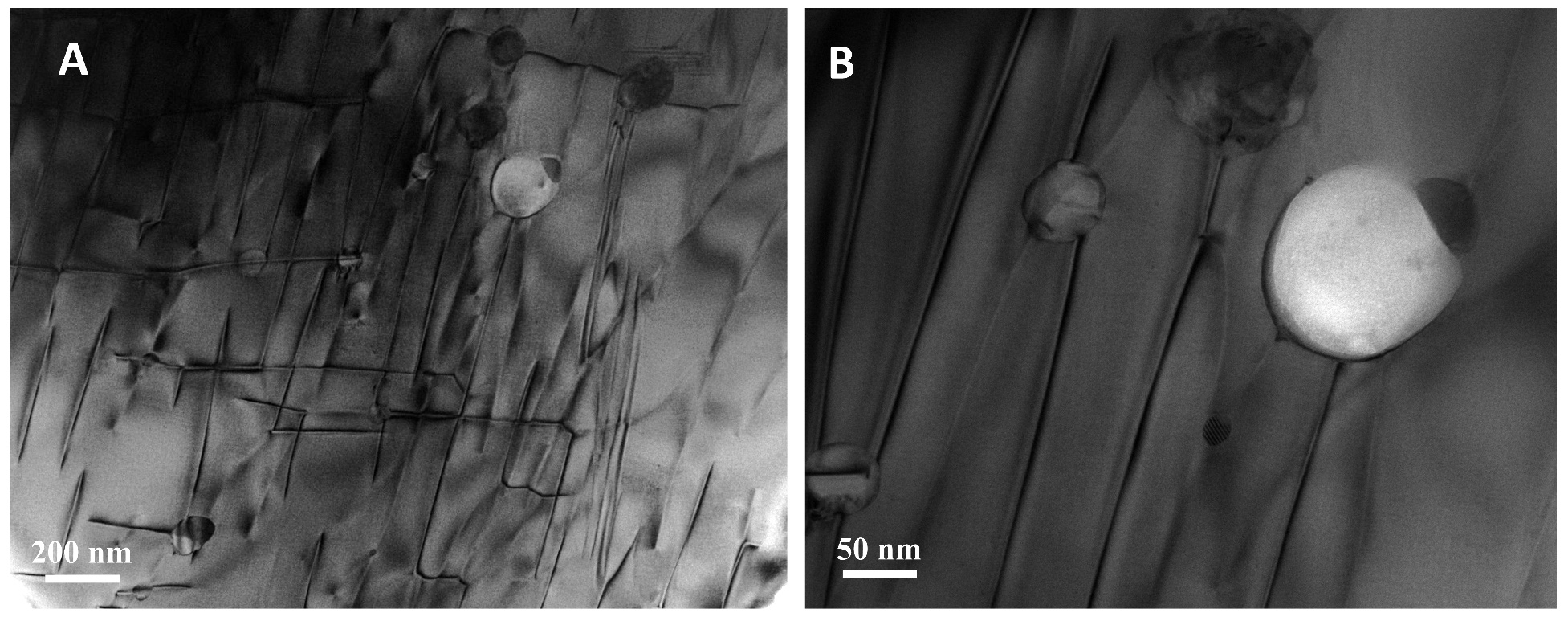


**Fig. S8** TEM image of the nanosecond-phase regions and dislocation in SnSe-Pb-Cl-2%Sn.


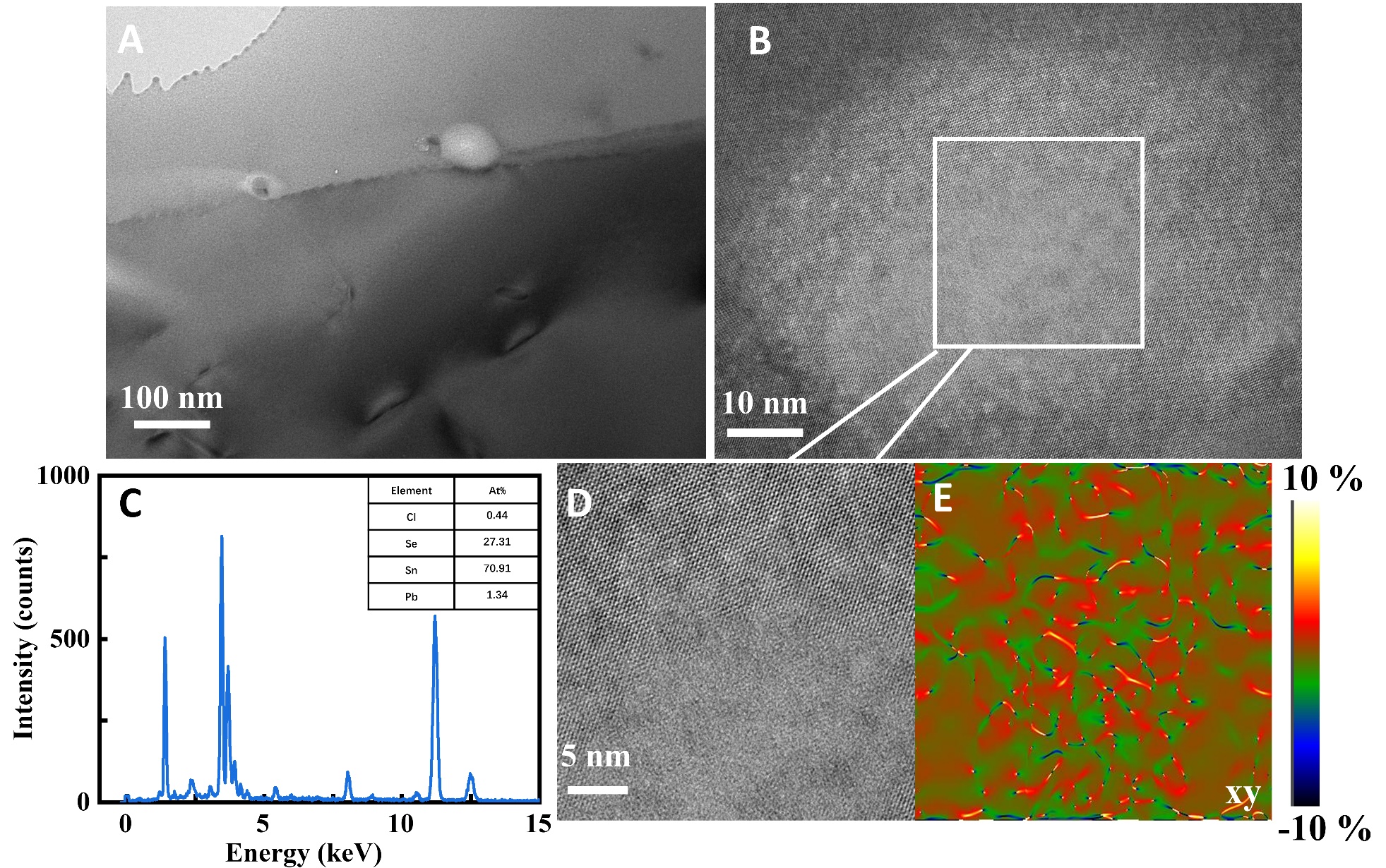


**Fig. S9** (A) TEM image, (B) and (D) HRTEM, (C) EDS, (E) GPA images the nano regions in SnSe-Pb-Cl-2%Sn.


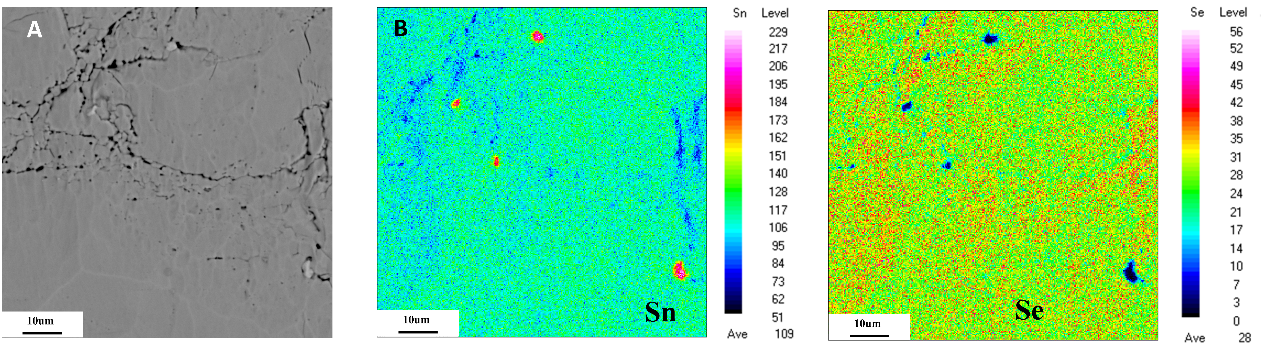


**Fig. S10** EPMA elemental mapping for Sn and Se in SnSe-Pb-Cl-2%Sn


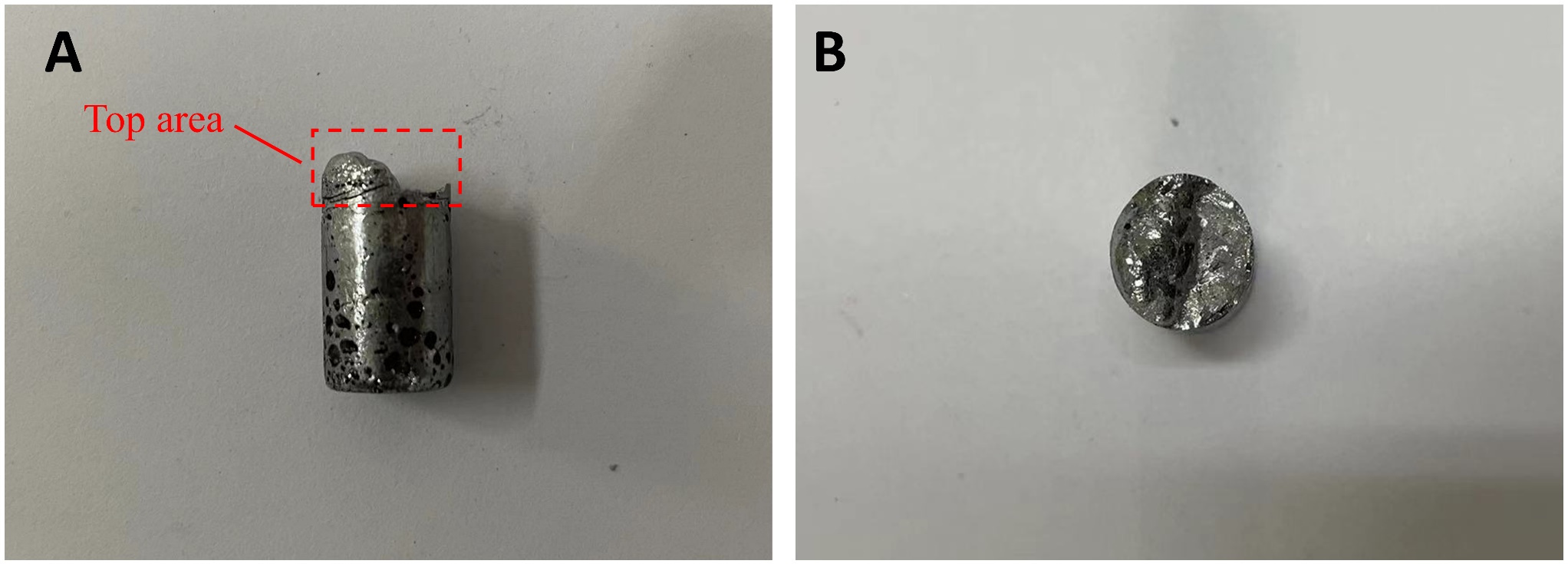


**Fig. S11** The morphology of the molten ingot for SnSe-Pb-Cl-2%Sn.


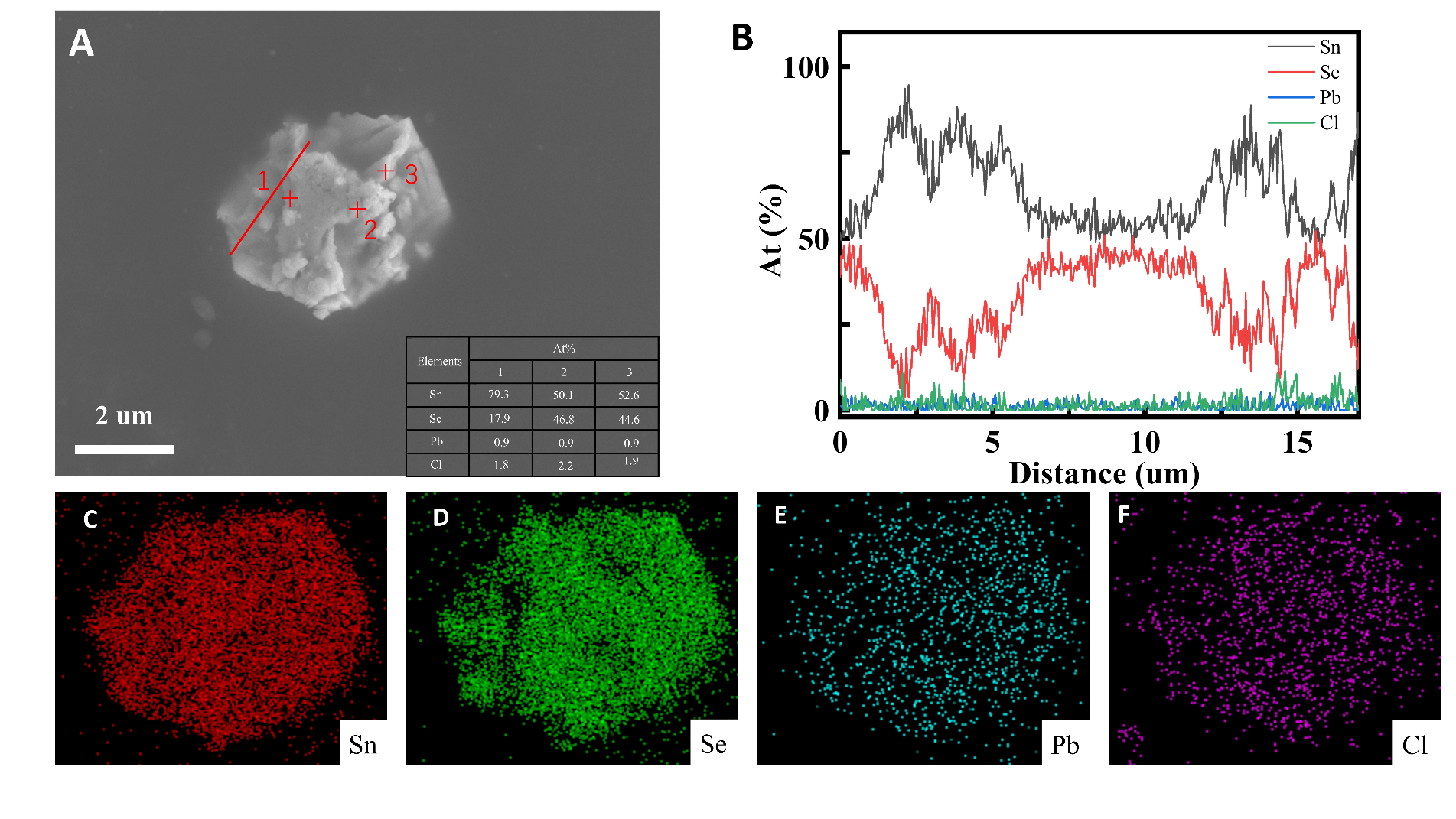


**Fig. S12** (A) SEM images, (B) EDS line scanning and (C)-(F) EDS maps of monotectic microplate in SnSe-Pb-Cl-2%Sn.


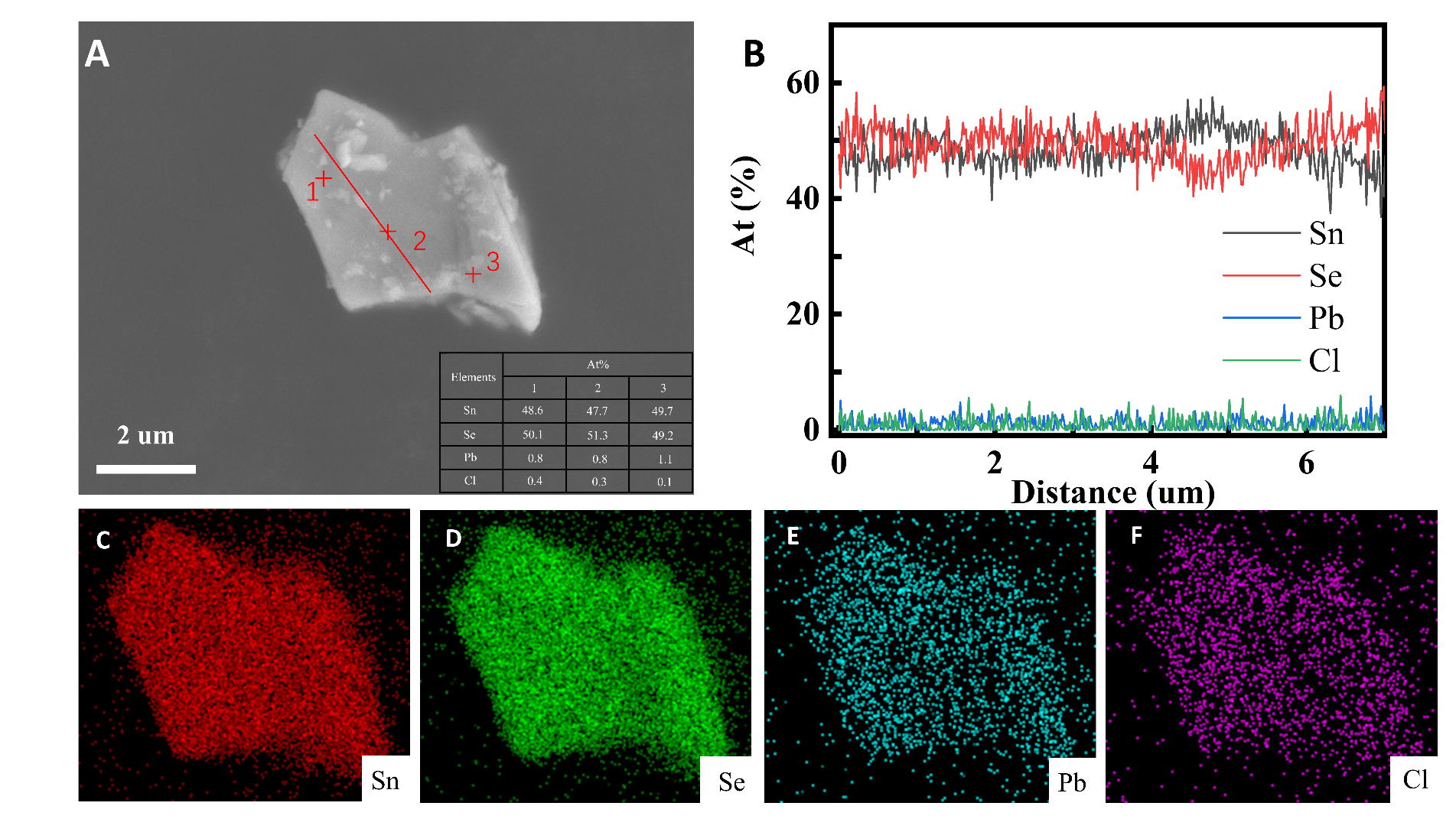


**Fig. S13** (A) SEM images, (B) EDS line scanning and (C)-(F) EDS maps of common microplate in SnSe-Pb-Cl-2%Sn.


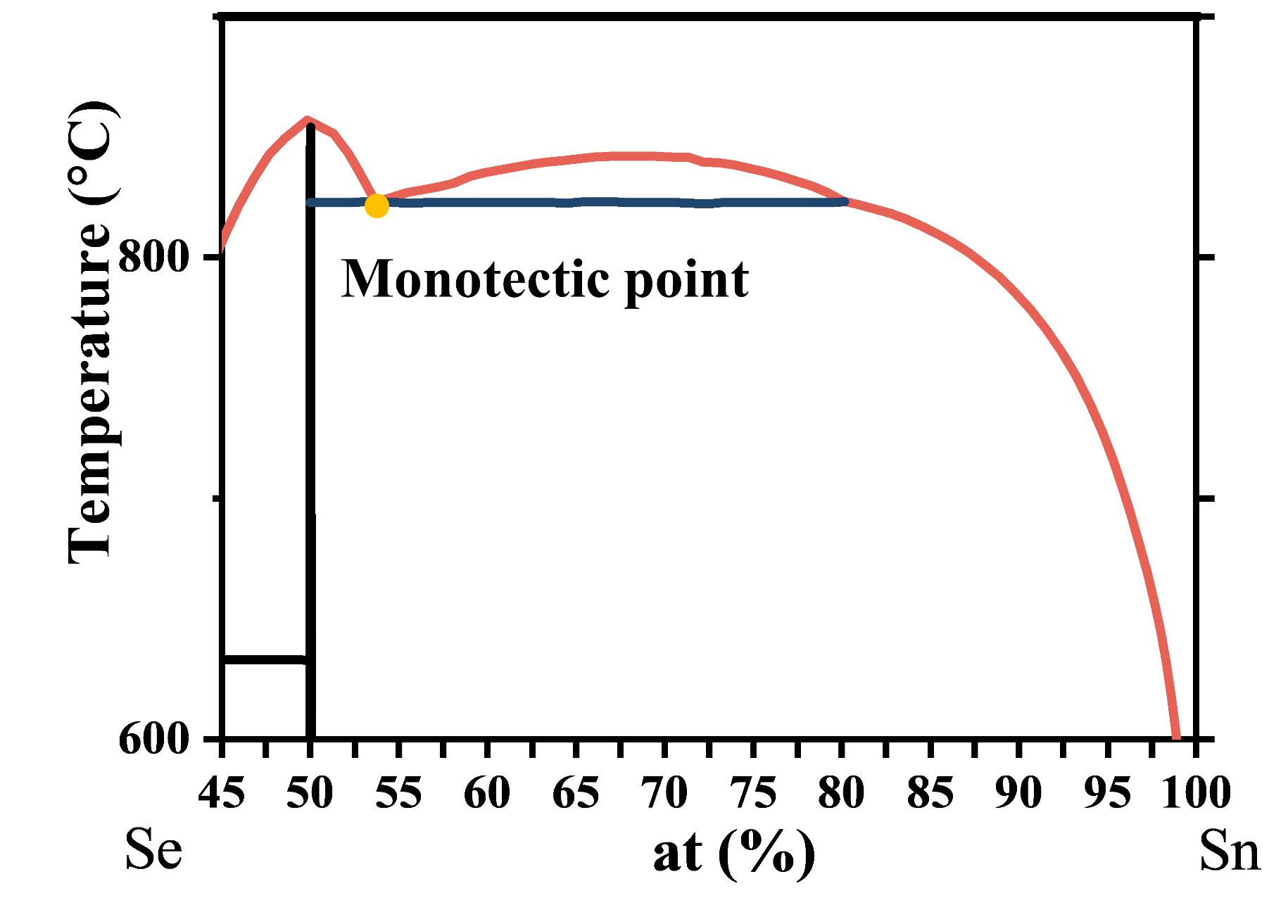


**Fig. S14** Phase diagram of SnSe system.


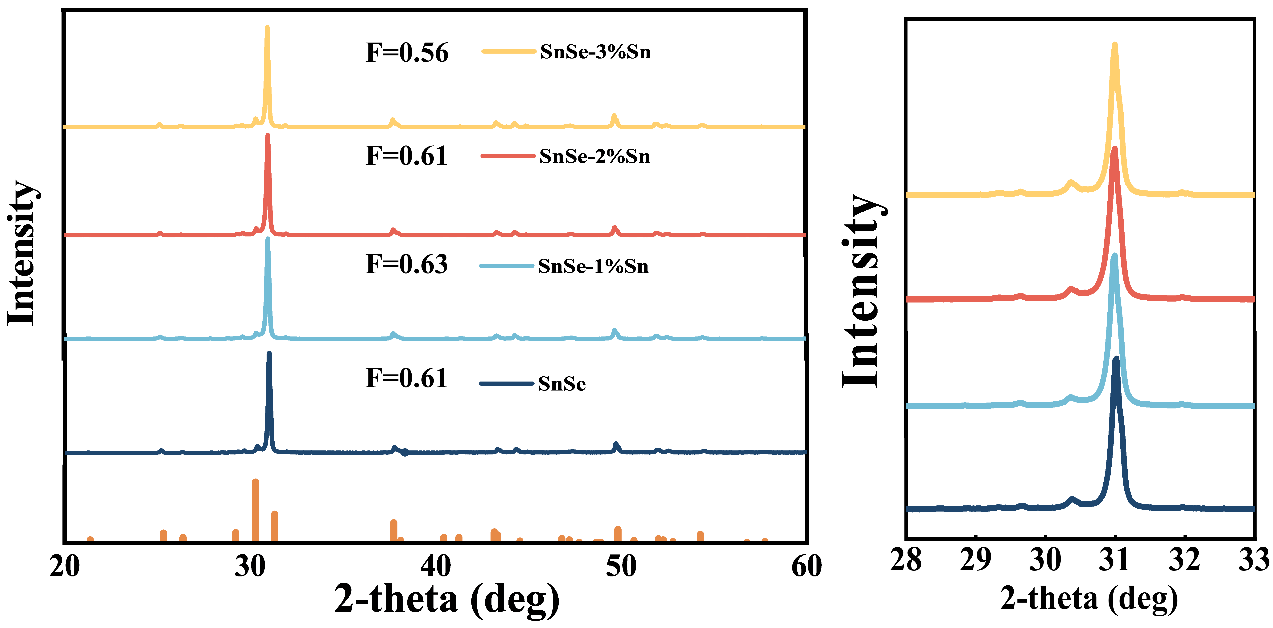


**Fig. S15** X-ray diffraction patterns of SnSe-Pb-Cl -x%Sn crushed by blade.


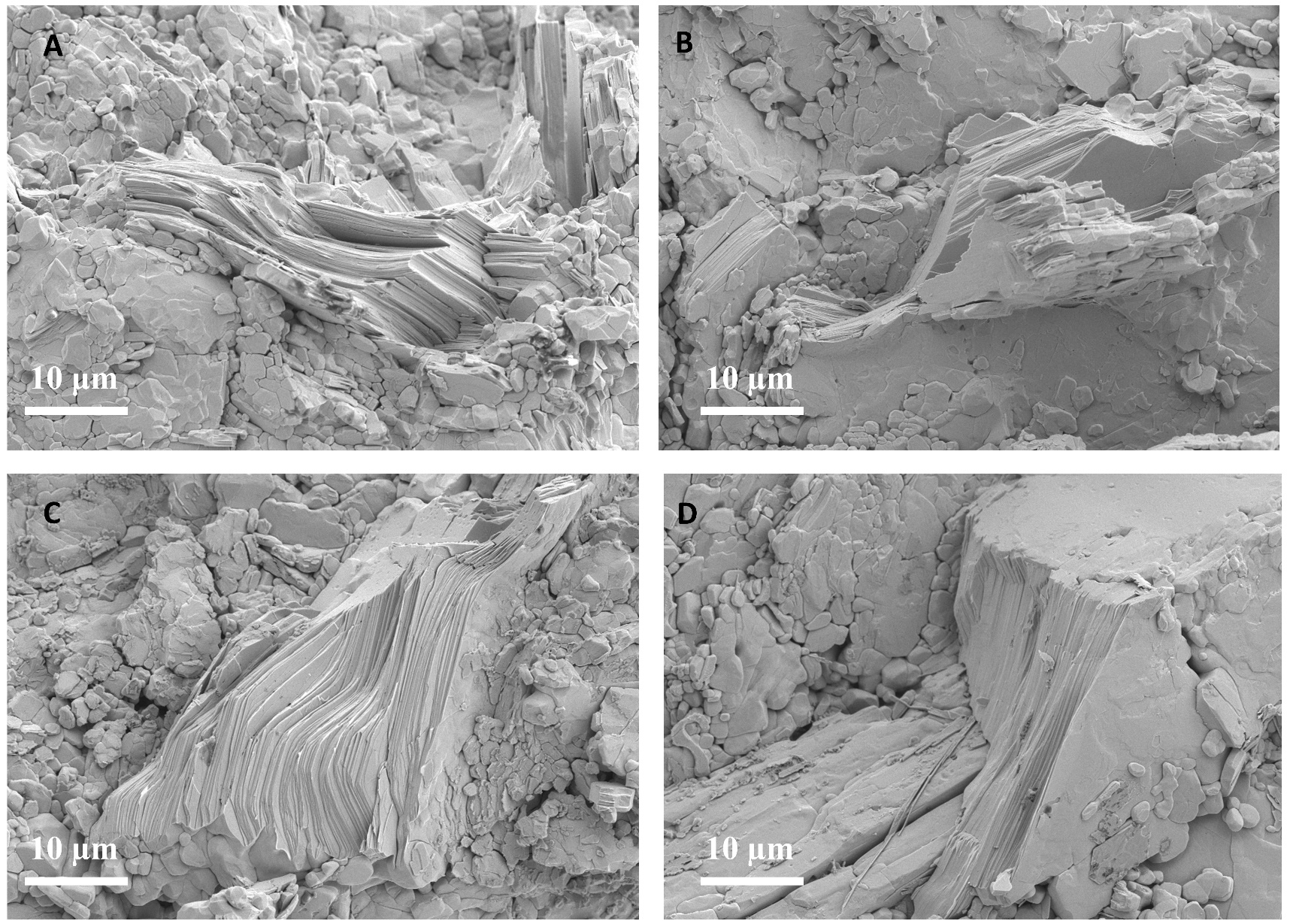


**Fig. S16** (A)-(D) SEM images of SnSe-Pb-Cl -x%Sn crushed by blade.


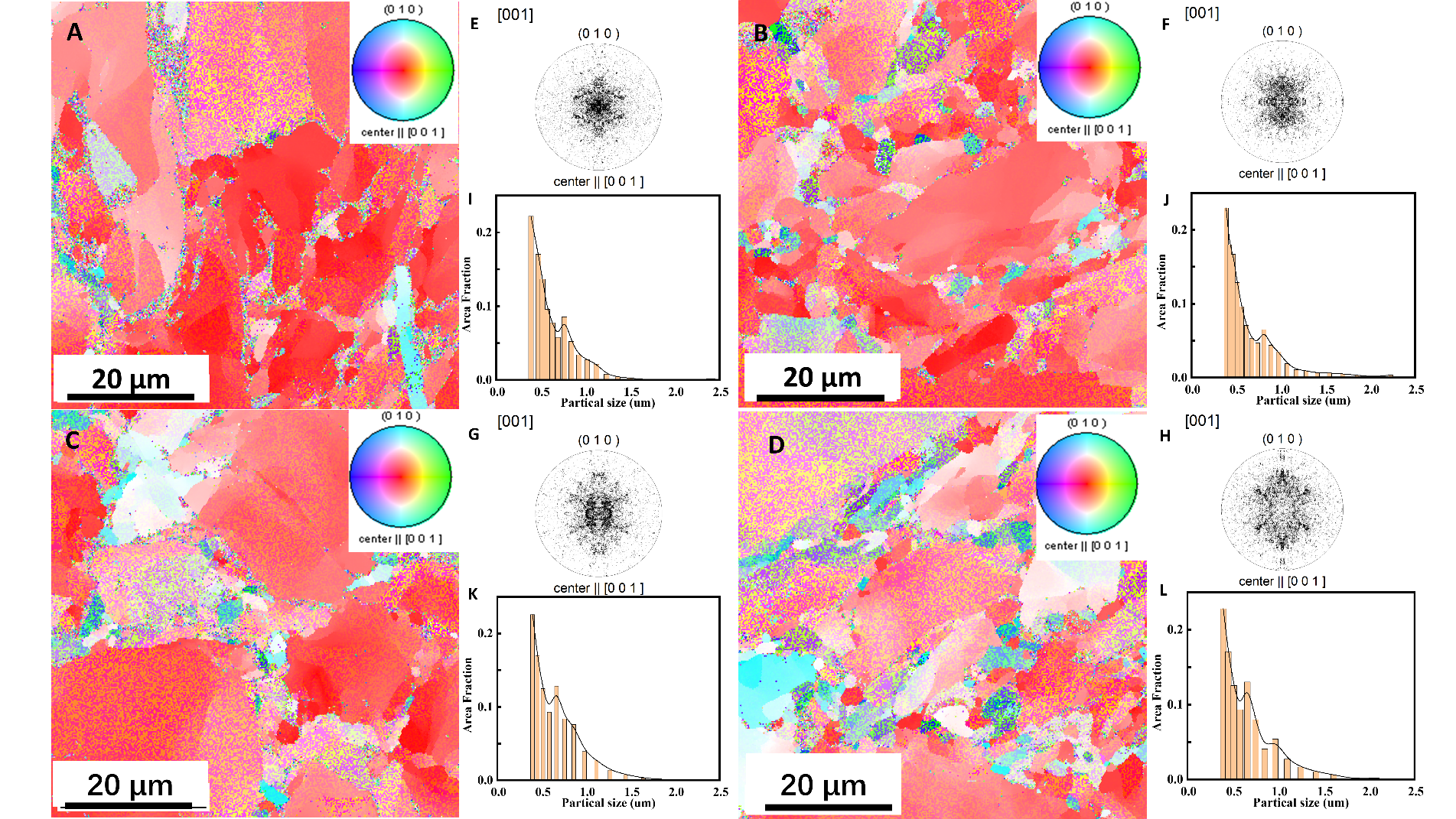


**Fig. S17** (A)-(D) EBSD images, (E)-(H) inverse pole figures and(I)-(L) grain size of SnSe-Pb-Cl -x%Sn.


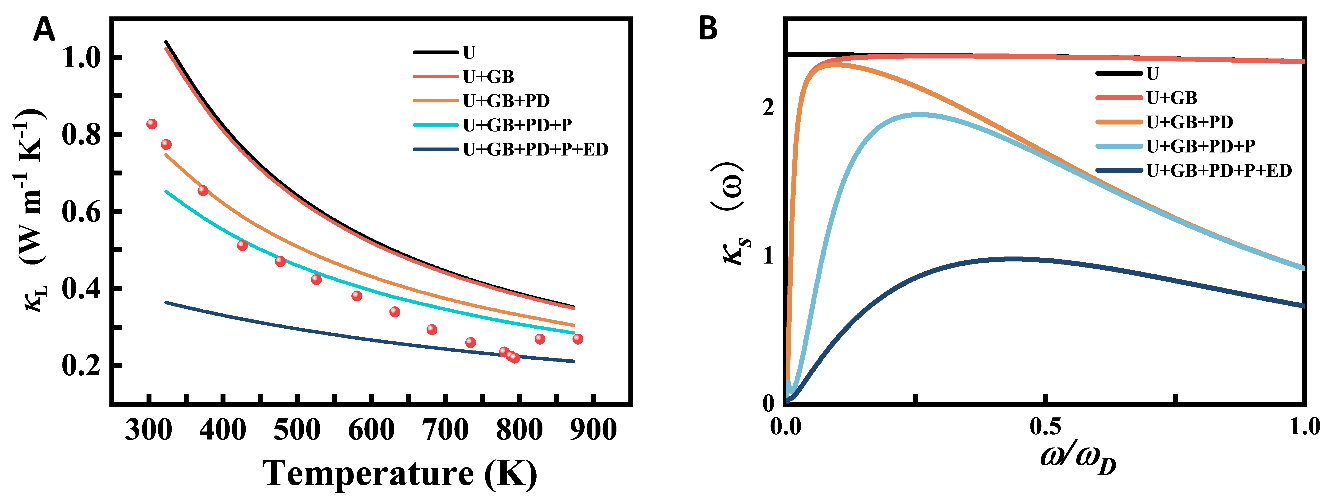


**Fig. S18** (A) The prediction lattice thermal conductivity of SnSe-Pb-Cl-x%Sn. Experimental *κ*_L_ for polycrystalline SnSe-2%Sn are included for comparison; the calculated *κ*_L_ using Debye-Callaway model; (B) Calculated *κ*_s_ using Debye-Callaway model with different phonon scatterings of Umklapp processes (U), grain boundaries (GB), point defects (PD), edge dislocations (ED) and precipitates (P).


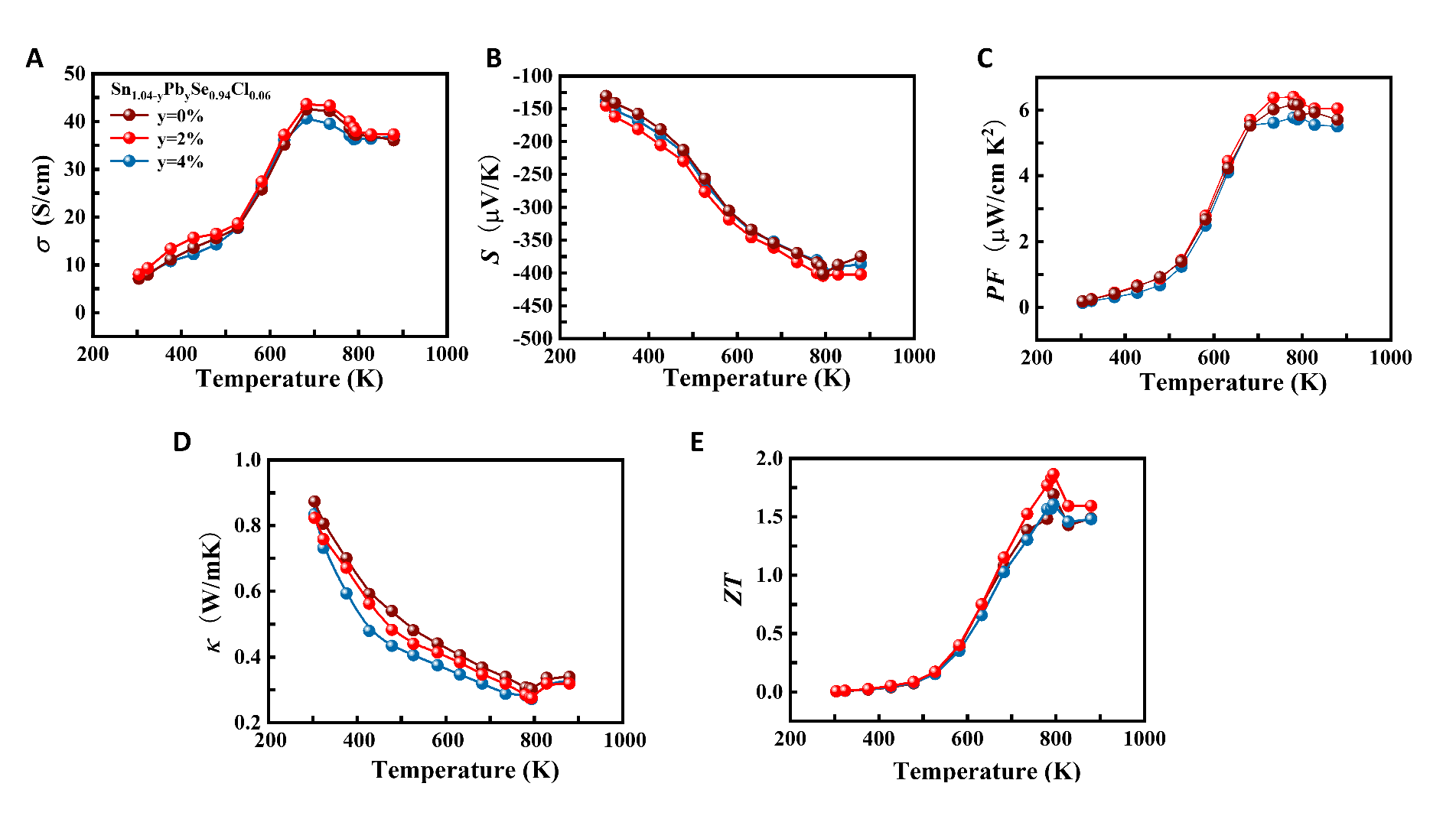


**Fig. S19** Temperature-dependent (A) *σ*, (B) *S* (C) *PF*, (D) *κ*_T_ and (E) *ZT* of Sn_1.04-y_Pb_y_Se_0.94_Cl_0.06_.

.


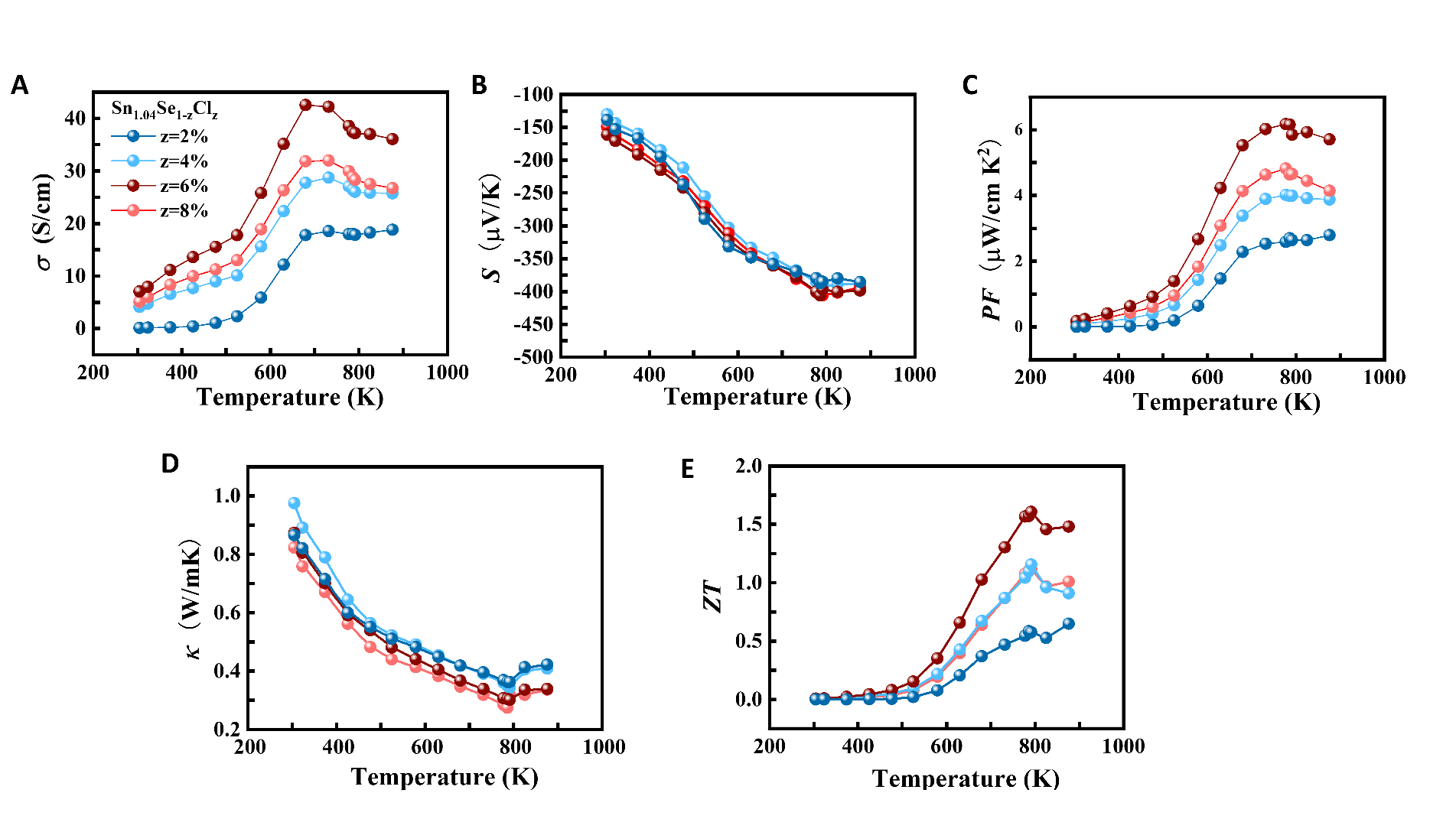


**Fig. S20** Temperature-dependent (A) *σ*, (B) *S* (C) *PF*, (D) *κ*_T_ and (E) *ZT* of Sn_1.04_Se₁₋_z_Cl_z_.

**Table. S1**. Apparent density of SnSe-Pb-Cl-x%Sn samples.

| Nominal compositions | Density (g/cm^3^) |
| --- | --- |
| SnSe-Pb-Cl | 5.97 |
| SnSe-Pb-Cl-1%Sn | 5.98 |
| SnSe-Pb-Cl -2%Sn | 5.90 |
| SnSe-Pb-Cl -3%Sn | 5.89 |

**Table. S2**. Nominal and actual compositions of SnSe-Pb-Cl-x%Sn (x=0, 1%, 2%, 3%).

| Sample | Sn | Se | Pb | Cl |
| --- | --- | --- | --- | --- |
| SnSe-Pb-Cl | 50.276 | 47.713 | 0.988 | 0.447 |
| SnSe-Pb-Cl-1%Sn | 51.012 | 48.136 | 1.029 | 0.400 |
| SnSe-Pb-Cl -2%Sn | 52.077 | 46.422 | 1.034 | 0.466 |
| SnSe-Pb-Cl -3%Sn | 53.607 | 45.614 | 1.054 | 0.438 |

**Phase Structure Characterization**

As shown in Fig.S11, when concentration of excess Sn is high (x≥2%), the molten ingot exhibits an unevenness. The majority of the ingot displays the characteristic dense and layered features of typical pure SnSe-Pb-Cl ingots, while the top part of the ingot formed loose polycrystalline. Then, the ingots particles in different area were observed by scanning electron microscopy (SEM) and energy dispersive spectrometer (EDS) (Fig.S12 and S13). The microplates located in the middle region are generally of a large size and possess a plate-like structure, with Sn and Se elements being evenly distributed. This suggests that the microplates are relatively homogeneous, and that there is no obvious secondary phase. The microplates located on top of the ingot, there are a great element fluctuation in the microplate observed by elements line scanning and mapping. The monotectic phase is gradually dispersed throughout the upper portion of the ingot as the temperature of the melt decreases, leading to the loose polycrystalline located on top area.

Fig. S15 shows the X-ray diffraction (XRD) patterns of sintered SnSe samples parallel to the SPS pressure direction. All diffraction peaks are well matched to the standard pattern of SnSe (PDF# 48-1224) and indexed to the orthorhombic structure with a space group of *Pnma* (#62). The incorporation of Pb^2+^ and Cl⁻ dopants introduce significant strain into the crystal structure, leading to lattice contraction, causing to higher angles compared to the standard reference. The texturing degree is then determined by the orientation factors, *F* using the following equations: ^[1]^

| $F\text{=}\frac{P-P_{0}}{1-P_{0}}$ | (S1) |  |
| --- | --- | --- |
| $P_{0}=\frac{I_{0}(h00)}{\sum I_{0}(hkl)}$ | (S2) |  |
| $P=\frac{I(h00)}{\sum I(hkl)}$ | (S3) |  |

where *P* and *P*_0_ are the ratio of integrated intensities of all (h00) planes to the intensities of all (hkl) planes for preferentially and randomly oriented samples. The *F* values of the samples are 0.61, 0.63, 0.62, and 0.56, respectively. Moreover, the morphologies of samples are clearly illustrated in the SEM images as shown in Fig. S16, the grains of the samples were clearly plate-like structure. According to the electron backscatter diffraction (EBSD) and inverse pole figure, the grain orientation of the sample along SPS pressure is mainly the (100) direction (Fig. S17). In addition, the grain size is invariable as the composition changes, indicating that liquid phase sintering does not have an effect on increasing the grain size of the material (Fig. S18I to L).

**Single parabolic model of electrical transport**

According to the single parabolic band model, thermoelectric properties are given by

Seebeck coefficient^[4,5]^

| $S\text{= }\frac{k_{B}}{e}\left( \frac{\left( \lambda+2 \right)F_{\lambda+1}\left( \eta\right)}{\left( \lambda+1 \right)F_{\lambda}\left( \eta\right)}-\eta\right)$ | (S4) |
| --- | --- |

Hall carrier concentration

| $n_{H}=\frac{8\pi(2m_{d}^{*}k_{B}T)^{3/2}}{3h^{3}}\frac{1+\lambda}{1/2+2\lambda}\frac{F_{\lambda}^{2}}{F_{2\lambda-1/2}}$ | (S5) |
| --- | --- |

in which

| $F_{n}\left( \eta\right)=\int_{0}^{\infty} \frac{\varepsilon^{n}}{1+e^{\varepsilon-\eta}}\text{ d}\chi$ | (S6) |
| --- | --- |

Electrical thermal conductivity is calculated according to the Wiedemann-Franz law ^[6]^

$\kappa_{e}=L\sigma T$ (S7)

where *L* represents the Lorentz number. *L* is given by

$L\text{= }\left( \frac{k_{B}}{e} \right)^{2}\left( \frac{\left( \lambda+3 \right)F_{\lambda+2}\left( \eta\right)}{\left( \lambda+1 \right)F_{\lambda}\left( \eta\right)}-\left[ \frac{\left( \lambda+2 \right)F_{\lambda+1}\left( \eta\right)}{\left( \lambda+1 \right)F_{\lambda}\left( \eta\right)} \right]^{2} \right)$ (S8)

where *k*_B_ is the Boltzmann constant, *e* is the electron charge, *S* is the Seebeck coefficient, *n*_H_ is the carrier concentration, and *λ* is the constant, respectively. *λ* is dependent on scattering factor *r*, which is equal to *r*+1/2. Assuming acoustic phonon scattering dominating the carrier scattering, *r* = – 1/2.

**Debye-Callaway’s model**

According to the Debye-Callaway model, the lattice thermal conductivity can be calculated from:^[7]^

| $\kappa_{L}=\frac{k_{B}}{2\pi^{2}\nu_{s}}\left( \frac{k_{B}T}{\hbar} \right)^{3}\int_{0}^{\theta_{D}/T} \tau_{\text{tot}}\frac{z^{4}e^{z}}{\left( e^{z}-1 \right)^{2}}dz$ | (S9) |
| --- | --- |

The integrand item in conjunction with the coefficient of Equation S6 is the spectral lattice thermal conductivity (*κ*_s_), namely:

| $\kappa_{s}=\frac{k_{B}}{2\pi^{2}\nu_{s}}\left( \frac{k_{B}T}{\hbar} \right)^{3}\tau_{tot}\frac{z^{4}e^{z}}{\left( e^{z}-1 \right)^{2}}$ | (S10) |
| --- | --- |

where *k*_B_ is the Boltzmann constant, *ν*_s_ is average sound speed, is the reduced Planck constant, *θ*_D_ is Debye temperature, *z* = *ℏω*/*k*_B_*T* (*ω* denoting the phonon frequency) is the reduced phonon frequency and *τ*_tot_ is total relaxation time, namely:

| $\tau_{\text{tot}}^{-1}=\tau_{U}^{-1}+\tau_{\mathrm{GB}}^{-1}+\tau_{\text{PD}}^{-1}+\tau_{\mathrm{DS}}^{-1}+\tau_{\text{SF}}^{-1}+\tau_{\text{TW}}^{-1}$ | (S11) |
| --- | --- |

Umklapp scattering process:

| $\tau_{U}^{-1}=\frac{\hbar\gamma^{2}}{\overline{M}\nu_{s}^{2}\theta_{D}}\omega^{2}T\exp\left( -\frac{\theta_{D}}{3T} \right)$ | (S12) |
| --- | --- |

Normal process:

| $\tau_{N}^{-1}=\beta\tau_{U}^{-1}$ | (S13) |
| --- | --- |
|  |  |

Grain boundaries scattering:

| $\tau_{\mathrm{GB}}^{-1}=\frac{\nu_{s}}{G}$ | (S14) |
| --- | --- |

Point defects scattering:

| $\tau_{\text{PD}}^{-1}=\frac{\overline{V}\omega^{4}}{4\pi{\nu_{s}}^{3}}\Gamma$ | (S15) |
| --- | --- |

Dislocation scattering:

| $\tau_{\mathrm{DS}}^{-1}=\tau_{\text{DC}}^{-1}+\tau_{\text{DE}}^{-1}$ | (S16) |
| --- | --- |
| $\tau_{\text{DC}}^{-1}=N_{D}\frac{V_{a}^{4/3}}{v_{a}^{2}}\omega^{3}$ | (S17) |
| $\tau_{\text{DE}}^{-1}=0.6\cdot B_{D}^{2}N_{D}\gamma^{2}\omega\left[ \frac{1}{2}+\frac{1}{24}\left( \frac{1-2\upsilon}{1-\upsilon} \right)\left( 1+\sqrt{2}\left( \frac{v_{l}}{v_{t}} \right)^{2} \right) \right]$ | (S18) |

Nano precipitates phonon scattering^[8,9]^:

| $\tau_{\text{np}}^{-1}=\upsilon\left[ \left( 2\pi R^{2} \right)^{-1}+\left( \frac{4}{9}\pi R^{2}\left( \frac{\Delta D}{D} \right)^{2}\left( \frac{\omega R}{\upsilon} \right)^{4} \right)^{-1} \right]^{-1}N_{p}$ | (S19) |
| --- | --- |

In the above equations, *γ* is the Grüneisen parameter, *β* is the ratio between normal process and Umklapp phonon scattering, *υ* is the Poison ratio, $\bar{V}$ is the average atomic volume, $\bar{M}$ is the average atomic mass, $\Gamma$is the point defect scattering parameter, *d* is the grain size, *a* is the lattice parameter, *B*_D_ is Burgers’ vector, and *N*_D_ is the density of dislocations. *D* and Δ*D* were calculated according to the theorical crystal structure.

**Supplementary Table 3**. Parameters for phonon modeling studies

| **Parameters** | **Values** |
| --- | --- |
| Debye temperature *Θ*_D_ (K) | 145 |
| Ratio of normal and Umklapp process *β* | 0.76 (fitted) |
| Longitudinal sound velocity *v*_L_ (m/s) | 2834 |
| Transverse sound velocity *v*_T_ (m/s) | 1550 |
| Sound velocity *v* (m/s) | 1674^[10]^ |
| Average atomic mass $\bar{M}$ (kg) | 1.97×10^-25^ |
| Average atomic volume $\bar{V}$ (m^3^) | 2.12×10^-29^ |
| Grain size *d* (μm) | 0.5 |
| Point defect scattering parameter *Γ* | 0.0653 (fitted) |
| Grüneisen parameter *γ* | 3.13^[11]^ |
| Lattice parameter *a，b，c* (Å) | 11.42 4.19 4.46 |
| Density of dislocation (cm^-2^) | 8×10^12^ |
| Magnitude of Burger’s vector *B*_D_ (Å) | 11.6 |
| Density *D* (g cm^-3^) | 6.2 |
| Density of precipitates *D*_2_ (g cm^-3^)  Number density of precipitates *N*_p_ (cm^-3^) | 7.28  200 |

**Reference**

[1] P.-P. Shang, J. Dong, J. Pei, F.-H. Sun, Y. Pan, H. Tang, B.-P. Zhang, L.-D. Zhao, J.-F. Li, *Research* **2019**, *2019*, 1.

[2] C. Chang, M. Wu, D. He, Y. Pei, C.-F. Wu, X. Wu, H. Yu, F. Zhu, K. Wang, Y. Chen, L. Huang, J.-F. Li, J. He, L.-D. Zhao, *Science* **2018**, *360*, 778.

[3] L. Su, D. Wang, S. Wang, B. Qin, Y. Wang, Y. Qin, Y. Jin, C. Chang, L.-D. Zhao, *Science* **2022**, *375*, 1385.

[4] S. Li, Z. Tong, H. Bao, *Journal of Applied Physics* **2019**, *126*, 025111.

[5] S. Johnsen, J. He, J. Androulakis, V. P. Dravid, I. Todorov, Duck. Y. Chung, M. G. Kanatzidis, *J. Am. Chem. Soc.* **2011**, *133*, 3460.

[6] L.-D. Zhao, S.-H. Lo, J. He, H. Li, K. Biswas, J. Androulakis, C.-I. Wu, T. P. Hogan, D.-Y. Chung, V. P. Dravid, M. G. Kanatzidis, *J. Am. Chem. Soc.* **2011**, *133*, 20476.

[7] J. Callaway, H. C. von Baeyer, *Phys. Rev.* **1960**, *120*, 1149.

[8] Z. Chen, Z. Jian, W. Li, Y. Chang, B. Ge, R. Hanus, J. Yang, Y. Chen, M. Huang, G. J. Snyder, Y. Pei, *Adv. Mater.* **2017**, *29*, 1606768.

[9] N. Mingo, D. Hauser, N. P. Kobayashi, M. Plissonnier, A. Shakouri, *Nano Lett.* **2009**, *9*, 711.

[10] L.-D. Zhao, S.-H. Lo, Y. Zhang, H. Sun, G. Tan, C. Uher, C. Wolverton, V. P. Dravid, M. G. Kanatzidis, *Nature* **2014**, *508*, 373.

[11] Y. Xiao, C. Chang, Y. Pei, D. Wu, K. Peng, X. Zhou, S. Gong, J. He, Y. Zhang, Z. Zeng, L.-D. Zhao, *Phys. Rev. B* **2016**, *94*, 125203.
